# Interactive effects of depth and differential irrigation on soil microbiome composition and functioning

**DOI:** 10.3389/frmbi.2023.1078024

**Published:** 2023-03-02

**Authors:** Dan Naylor, Katherine Naasko, Montana Smith, Sneha Couvillion, Carrie Nicora, Jesse Trejo, Steven Fransen, Robert Danczak, Ryan McClure, Kirsten S. Hofmockel, Janet K. Jansson

**Affiliations:** ^1^ Earth and Biological Sciences Directorate, Pacific Northwest National Laboratory, Richland, WA, United States; ^2^ Department of Crop and Soil Sciences, Washington State University, Pullman, WA, United States; ^3^ Environmental Molecular Sciences Laboratory, Pacific Northwest National Laboratory, Richland, WA, United States; ^4^ Department of Crop and Soil Sciences, Washington State University, Prosser, WA, United States; ^5^ Department of Agronomy, Iowa State University, Ames, IA, United States

**Keywords:** microbiome, irrigation, soil depth, rhizosphere, plant-microbe interactions, amplicon sequencing, transcriptomics, soil metabolomics

## Abstract

Two factors that are well-known to influence soil microbiomes are the depth of the soil as well as the level of moisture. Previous works have demonstrated that climate change will increase the incidence of drought in soils, but it is unknown how fluctuations in moisture availability affect soil microbiome composition and functioning down the depth profile. Here, we investigated soil and wheatgrass rhizosphere microbiomes in a single common field setting under four different levels of irrigation (100%, 75%, 50%, and 25%) and three depths (0-5 cm, 5-15 cm, and 15-25 cm from the surface). We demonstrated that there is a significant interactive effect between depth and irrigation, where changes in soil moisture more strongly affect soil microbiomes at the surface layer than at deeper layers. This was true for not only microbiome community composition and diversity metrics, but also for functional profiles (transcriptomic and metabolomic datasets). Meanwhile, in rhizosphere communities the influence of irrigation was similar across the different depths. However, for the ‘Alkar’ wheatgrass cultivar, the rhizosphere microbial communities responded more strongly to changes in irrigation level than did the communities for the ‘Jose’ cultivar rhizosphere. The lessened response of deeper soil microbiomes to changes in irrigation may be due to higher incidence of slow-growing, stress-resistant microbes. These results demonstrate that the soil microbiome response to moisture content is depth-dependent. As such, it will be optimal for soil microbiome studies to incorporate deeper as well as surface soils, to get a more accurate picture of the soil microbiome response to stress.

## 1 Introduction

The soil microbiome is linked to numerous ecological processes, including nutrient cycling, gas exchange, carbon sequestration, and bioremediation (Amundson et al., 2015; Jacoby et al., 2017). Unfortunately, such ecosystem functions are increasingly under threat from uncertain moisture availability due to climate change. In particular, drought stress presents a significant challenge to the soil microbiome, threatening microbial interactions (de Vries et al., 2018), enzymatic activity and respiration (Curiel Yuste et al., 2007; Borowik and Wyszkowska, 2016), substrate supply (Tecon and Or, 2017), biomass levels (Rangel-Vasconcelos et al., 2015), and community composition (Naylor and Coleman-Derr, 2018). In addition, the soil microbiome is linked to plant health (Wardle, 2004; Lakshmanan et al., 2014) through activities like phytohormone production and nutrient solubilization (Song et al., 2020), so stresses such as drought that impact the soil microbiome may compound the negative implications for plant life.

While the effects of moisture availability on the microbiome have been extensively studied (Naylor and Coleman-Derr, 2018), there remains a significant knowledge gap as to how these effects vary across different soil depths. The majority of soil microbiome studies to date have focused on surface soils (Eilers et al., 2012), which tend to sample only the top 5-10 cm (Bachar et al., 2010; Felsmann et al., 2015; Bouskill et al., 2016). Such studies may neglect differentiation between surface and deeper levels, with respect to factors such as chemical concentrations (Salome et al., 2010; Bai et al., 2017), physical structure (Rumpel et al., 2012), gas levels (Chirinda et al., 2014), and moisture content (Yan et al., 2017). Variations in soil physiochemistry with depth tend to be reflected by changes in the microbiome, typically including decreases in deeper soils for soil microbial diversity (Brewer et al., 2019), biomass (Spohn et al., 2016; Young et al., 2019), and total activity (Ko et al., 2017; van Leeuwen et al., 2017). Deeper soil microbiomes are taxonomically (Eilers et al., 2012; Bai et al., 2017; Seuradge et al., 2017; Tripathi et al., 2019) and functionally (Zhang et al., 2017; Young et al., 2019) distinct from those at the surface – for instance, microbes respond to deep soil conditions by activating stress and starvation response pathways, decreasing enzymatic activity for non-essential processes, favoring pathways for oligotrophic carbon metabolism, or else largely localizing around the few carbon-rich hotspots such as plant roots (Naylor et al., 2022). Despite lower overall activity, subsoil microbes contribute substantially to processes like nutrient cycling (Schlatter et al., 2018; Tang et al., 2018), soil formation (Buss et al., 2005), and carbon turnover (Lomander et al., 1998; de Sosa et al., 2018). However, it remains to be seen how changes in irrigation affect microbes at different depths of the rhizosphere (the region of soil immediately surrounding the root and that which is subject to the plant’s influence (Hartmann et al., 2008; Bakker et al., 2013) – for instance, whether or not microbiome changes in the surrounding bulk soil are mirrored in the rhizosphere, or else circumvented by the plant’s influence.

Changes in soil moisture due to differential precipitation or irrigation can influence soil microbial composition and functioning, including favoring oligotrophic taxa and microbes with thicker cell walls, decreasing nutrient cycling processes, or increasing their osmolyte production (Naylor and Coleman-Derr, 2018). The few studies that have considered the interaction between moisture and depth have found little to no significant interactive effects on the soil microbiome composition (Hartmann et al., 2017; Upton et al., 2020), although it has been shown that specific microbial phyla vary in how they respond to irrigation across different soil depths (Chodak et al., 2015; Hartmann et al., 2017). As different microbial taxa carry out distinct ecosystem functions, understanding the interaction between soil moisture and depth will shed light on how fluctuating moisture will affect soil functioning across different soil layers.

Here, we aimed to test how irrigation affects the composition and potential functions of the soil microbiome at different soil depths. First, we hypothesized that the effects of irrigation would be less pronounced at lower soil depths. We believed this to be the case as there would be less water available and microbes in deeper soils are already adapted to stressful conditions (Jiao et al., 2018; Brewer et al., 2019) and thus more resistant to water limitation. Alternatively, deeper soils are better-insulated from fluctuations in water availability than are surface soils. We anticipated that the microbial communities present at lower soil depths, or under low-moisture conditions, would have higher abundances of slow-growing and/or oligotrophic groups. Second, we hypothesized that rhizosphere microbiomes would not be as strongly impacted by irrigation and/or soil depth as those residing in bulk soils. Plants recruit a specific, tailored microbiome that is less diverse than the surrounding soil, especially under stressful conditions such as drought (Hartman and Tringe, 2019). Therefore, as the plant maintains tight control over the rhizosphere community, its influence will likely overpower the influence of soil moisture content. We further proposed that genotypic differences in plants will also influence the rhizosphere response, as rhizosphere microbiome responses to drought treatment have been shown to be distinct between different plant species or even cultivars of the same species (Naylor et al., 2017). Finally, we hypothesized that both soil moisture and depth would be significant determinants of the soil microbiome’s functional potential. It should be noted that we chose to focus on bacteria, omitting the fungal community, given that the fungal response to drought tends to be small (Barnard et al., 2013; Fuchslueger et al., 2016). Likewise, the fungal community composition does not tend to vary much with depth (Jumpponen et al., 2010; Hao et al., 2020), possibly as hyphal growth allows fungi to be physically present at multiple soil depths at the same time.

To test these hypotheses, we investigated changes in soil and rhizosphere microbiomes across different watering regimes and soil depth in an irrigated field trial, including three timepoints that together spanned the full field season. We obtained samples from a field site that represents naturally arid, but artificially irrigated, marginal land. This field site was previously planted with two cultivars of the bioenergy feedstock ‘tall wheatgrass’ (*Thinopyrum ponticum*) (Scheinost et al., 2008). Our experimental design and multi-omics approach allowed us to investigate the effects of irrigation, depth, and their interactions on both the soil microbiome and its functional response (metatranscriptome and metabolome). In addition, we were able to describe the differences between rhizosphere and bulk soil responses, as well as the influence of wheatgrass cultivars on the soil microbiome.

## 2 Methods

### 2.1 Field trial setup

The field site used in this study was located at the Washington State University Irrigated Agriculture Research and Extension Center (WSU IAREC) located in Prosser, WA. All soils were collected from the exact same field site. Soils from this site are in the Warden silt loam series, have an alkaline pH ~8, and are arid with a low organic matter content, as detailed previously (Zegeye et al., 2019). At this field site, a stand of tall wheatgrass (*Thinopyrum ponticum*) was established in May 2018. In this stand, there were 60 plots in a randomized complete block design (**Supplemental Figure 1**), with three replications and two subsample variety plots per irrigation treatment. This field site was established to investigate the effects of differential irrigation on tall wheatgrass growth- and yield-associated phenotypes. Plants were cut down (here, referred to as ‘harvesting’) at two points during the season (mid-July and late October 2020) to collect plant biomass and measure phenotypes. Watering was done as relative to established levels of tall wheatgrass crop consumptive water use (i.e. the level of evapotranspiration, or ETc, inherent to tall wheatgrass). Five irrigation lines supplied water to the following treatments: T1 = 100% ETc, T2 = 75% ETc, T3 = 50% ETc, T4 = 25% ETc, and an unplanted treatment with 25% ETc on bare soil (samples from this treatment were not used in our experiment). On 7/20/2020, the strength of the drought treatments was increased to heighten the phenotypic effects of differential irrigation: T1 remained at 100% ETc, whereas T2 changed from 75% to 56.25% ETc, T2 changed from 50% to 37.5% ETc, and T4 changed from 25% to 18.75% ETc. Prior to the field season, all lines were irrigated at 100% normal field application rates until 4/28/2020, after which point differential irrigation treatments were put in place.

Two cultivars of perennial tall wheatgrass were used in the field trial: ‘Alkar’ and ‘Jose’. Alkar is widespread in the northwest of the North American Continent. It has been bred for soil reclamation on salty, alkaline soils, and is well-adapted to irrigated or partially irrigated sites (Scheinost et al., 2008), such as our field site in Eastern Washington state. The Jose cultivar has been adapted to grow in the southwest USA and also grows well in alkaline or saline soils (Scheinost et al., 2008). Both cultivars of tall wheatgrass are also potential bioenergy feedstocks that are well-adapted to growth on marginal land and capable of withstanding drought stress.

### 2.2 Sampling setup

**Figure 1:**
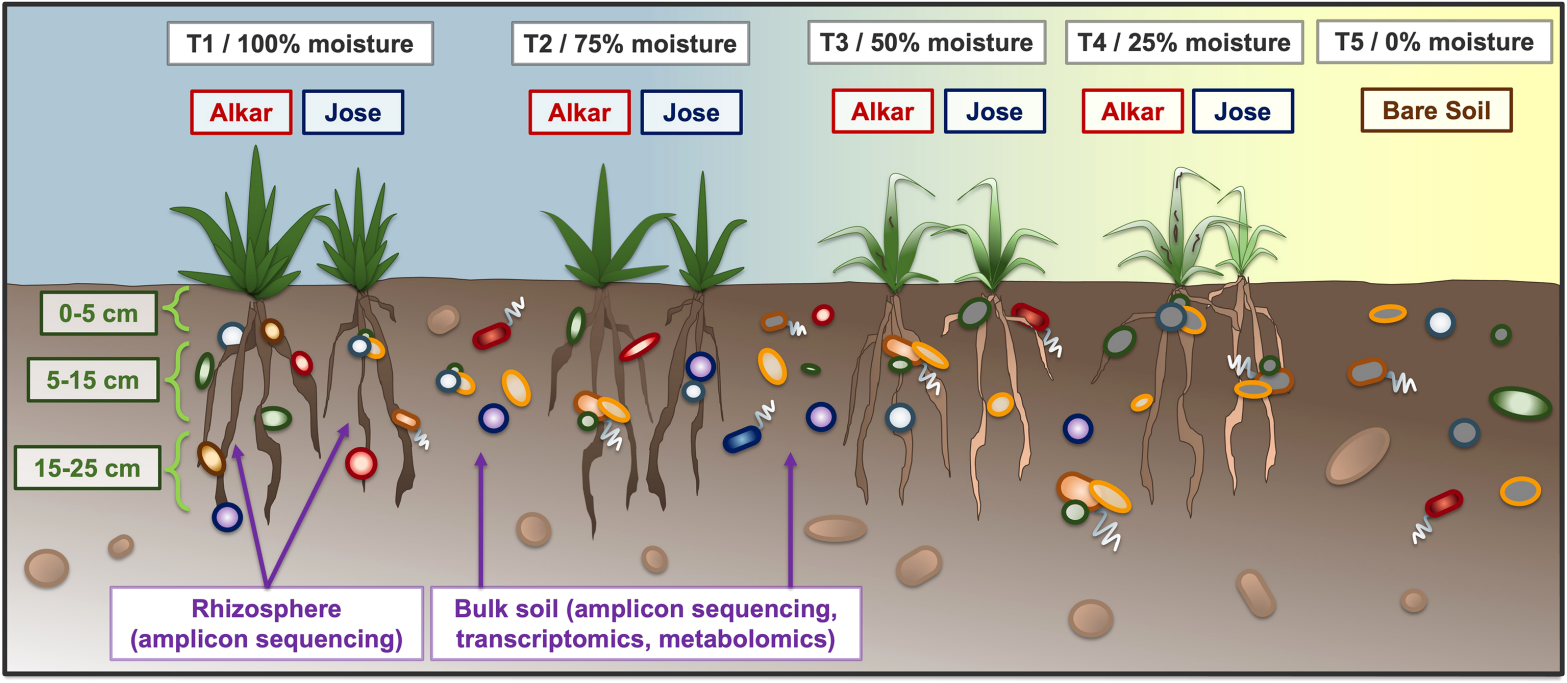
Schematic of the field experiment. Two cultivars of tall wheatgrass (‘Alkar’ and ‘Jose’) were planted in a common field, and grown under four irrigation regimes ranging from full moisture (T1, 100%) to very little moisture (T4, 25%). Samples were collected from three depth intervals: 0-5 cm, 5-15 cm, and 15-25 cm, which was roughly as deep as the wheatgrass roots could penetrate. Sample types taken included both rhizosphere and bulk soil, which were then used for amplicon sequencing as well as (bulk soil only) transcriptomics and metabolomics analyses. Non-irrigated soils (T5) were taken as a control.

Soil samples were collected on three sampling dates: 4/29/2020 (immediately prior to the start of differential irrigation treatments), 7/7/2020 (at the conclusion of the first treatment and soon before the initial aboveground plant harvest), and 10/15/2020 (at the conclusion of the second treatment, and soon before the final aboveground plant harvest). Bulk soil samples were collected from 27 plots, representing 4 irrigation treatments (from most to least irrigated: T1, T2, T3, T4), 2 wheatgrass cultivars (Alkar and Jose), and 3 biological replicates, along with 3 unplanted, non-irrigated (T5) sites immediately adjacent to the plots to represent soil controls (**Supplemental Figure 1**). The experimental design is summed up in **Figure 1**.

At each plot, 4 replicate soil cores were collected using a 36” long, ¾” diameter coring device. Before and between sampling, the coring device was washed and sterilized with 70% ethanol. The cores were collected approximately 6 inches from the nearest plant. Using a ruler, three depth sections were taken from each of the 4 cores, representing the depths (relative to the surface) 0-5 cm, 5-15 cm, and 15-25 cm. The 4 replicates of each depth section per plot were then pooled together, resulting in a weight of approximately 70 – 100 g soil for each depth sample per plot. Together this resulted in a total of 81 soil samples per sampling date and 243 total samples. Each soil sample was stored in a sterile aluminum foil bag and kept on ice for up to 6 h before returning to the laboratory, at which point they were flash-frozen in liquid N_2_ and stored at -80°C.

For the July and October sampling dates, rhizosphere samples were taken from the same plots as for the bulk soil samples (exempting the unplanted control samples). Rhizosphere soils (defined as those still closely associated with the roots after loosely-adhering soils are removed) were collected in a manner consistent with previous studies (Coleman-Derr et al., 2016; Naylor et al., 2017; Simmons et al., 2018). Root systems were dug up, shaken gently to remove loose soil and other debris, then were sectioned by the same depth increments as for the soil and placed into Falcon tubes containing epiphyte removal buffer (0.75% KH_2_PO_4_, 0.95% K_2_HPO_4_, 1% Tween-20 in sterile ddH_2_O, filter-sterilized at 0.2 μm) (Desgarennes et al., 2014). Tubes were stored on ice after harvest and during ~1 h transport to the laboratory. The samples were immediately thoroughly vortexed to allow for soil remaining on the roots (the ‘rhizosphere’ soil) to become loose and float to the bottom of the tube. The now-’clean’ roots were removed from the tubes and disposed of, while the tubes were centrifuged 5 min. at 6000 *x*g to pellet the rhizosphere soil. The supernatant was decanted and the soil was transferred into cryovials before flash-freezing in liquid N_2_.

Soil moisture content was calculated using the gravimetric method, in which ~5-6 g of soil from each sample was weighed, dried in an oven at 60°C for 3 days, then reweighed. Moisture content was quantified by subtracting 1 from the ratio of the original measurement to the final measurement.

### 2.3 16S rRNA gene amplicon library preparation and sequencing

Genomic DNA was extracted from soil and rhizosphere samples using the Zymo *Quick*-DNA fecal/soil microbe miniprep kit (catalog no. D6010) according to the manufacturer’s instructions (Zymo Research; Irvine, CA) with the modification of eluting in 100 uL elution buffer. Sample concentrations were quantified using the Qubit dsDNA HS assay kit (Thermo Fisher). For rhizosphere samples only, DNA was subsequently purified using Zymo’s ZR-96 DNA Clean and Concentrator kit (catalog no. D4024) to account for the low DNA concentrations of these samples.

Sequencing was performed as described previously (Naylor et al., 2020a). Sequences were amplified on the MiSeq platform (Illumina, San Diego, CA) using 16S primers (515F and 806R) specific to the V4 region. Raw sequence data was processed with default parameters using the pipeline Hundo (Brown et al., 2018), an in-house protocol that wraps multiple programs (BBDuk, VSEARCH, FastTree2) for amplicon quality control and annotation against the Silva database. The resulting data object consisted of 7,437,824 reads across 372 samples; after using the rarefy_even_depth function in the package ‘phyloseq’ (version 1.40.0) (McMurdie and Holmes, 2013) to obtain an even depth of 3,750 reads per sample, this was reduced to 1,353,750 reads across 361 samples. Downstream statistical analyses on 16S datasets were performed using the program R and the packages ‘phyloseq’ (version 1.40.0) (McMurdie and Holmes, 2013) and ‘vegan’ (version 2.6.4) (Oksanen et al., 2019). Other functions used included the aov function from the ‘stats’ package (version 4.2.1) (R Core Team, 2019) for ANOVA, the individual function from package ‘agricolae’ (version 1.3.5) (de Mendiburu, 2019) for indicator species analysis, the adonis2 function from ‘vegan’ (version 2.6.4) (Oksanen et al., 2019) for principal coordinate analysis, and the results function from ‘DESeq2’ (version 1.36.0) (Love et al., 2014) for DESeq2 enrichment tests. Statistical tests were conducted using default parameters, apart from polyserial analyses (which used the polyserial function from package ‘polycor’ (version 0.8.1) (Fox and Dusa, 2022) which used a maximum-likelihood estimate rather than the default two-step approximation, so as to increase the accuracy of the result. Normality of the data was tested using function ‘shapiro.test’, again from the stats package, confirming that our data did not follow a normal distribution (however, our ANOVA tests were one-way, which is robust to deviations from normality and thus can be used regardless of the data’s distribution). We confirmed initial ANOVA results using Kruskal-Wallis tests (a non-parametric method, where one-way ANOVA tests can be done on non-normal data). More information on the code, functions and packages used, can be found at www.github.com/dtnaylor/SoilDepth.

### 2.4 RNA library generation and sequencing

A subset of samples from the October timepoint (0-5 cm and 15-25 cm depths, with T1, T4, and control irrigation (T5) regimes) were chosen for RNA sequencing. Total RNA was extracted using the Zymo *Quick*-RNA fecal/soil microbe miniprep (catalog no. R2040), incorporating the DNase I treatment using Zymo’s DNase I kit (catalog no. E1010). To increase the yield of RNA, we modified the manufacturer’s instructions by first doubling the amount of soil per extraction (from 0.25 g to 0.5 g) and by performing extractions in triplicate before pooling separate extractions together. Certain soil samples (largely those from deeper soil layers) had low yield (< 100 ng per extraction) so additional rounds of extraction were performed to obtain sufficient RNA. RNA concentration was assessed using a Qubit RNA HS assay kit (Thermo Fisher) and RNA quality was determined using an Agilent 2100 BioAnalyzer (Agilent; Santa Clara, CA). The resultant RNA samples were then sequenced by GENEWIZ using Illumina technology (GENEWIZ; South Plainfield, NJ). Sequences were then aligned to a soil metagenome previously obtained from the same site (Nelson et al., 2020) using the Burrows-Wheeler aligner (BWA) (Li and Durbin, 2010). SAM files were then converted to raw counts using HTSeq (Anders et al., 2015).

Downstream analyses of the transcriptomic dataset were performed in R using the packages ‘DESeq2’ (Love et al., 2014) and custom scripts also available on GitHub. The ‘keggGet’ function from the ‘KEGGREST’ (Tenenbaum and Bioconductor Package Maintainer, 2021) package was used (under default parameters) to add annotations to the KO’s, including BRITE pathway information. To determine functional enrichment for a given pathway, Fisher’s exact test was used to see whether the pathway was disproportionately represented in the transcripts for a particular factor level relative to its representation in the whole dataset. iPath 3.0 was used to visualize KO enrichment patterns on a microbial metabolic map (Darzi et al., 2018).

### 2.5 Soil metabolomics

A subset of samples was chosen for metabolomics analysis (all depths, for all timepoints, but only the irrigation extremes (T1 and T4 irrigation regimes) along with unirrigated controls (T5) soils). A 10 g portion of each soil sample was aliquoted into 50 mL conical centrifuge tubes (Genesee Scientific, catalog no. 28-108; San Diego, CA). Added to these tubes were 10 mL of 0.9–2.0mm stainless steel beads, 0.1mm zirconia beads and 0.1mm garnet beads (Next Advance, Troy, NY). All beads had previously been washed with chloroform and methanol and dried in a fume hood. Extraction was performed using the Soil MPLex extraction protocol as previously described (Nicora et al., 2018).

GC-MS raw data file processing was done using Metabolite Detector software, including chromatographic alignment of all datasets (Hiller et al., 2009), and metabolites were identified by matching experimental spectra and retention indices to an augmented version of FiehnLib (Kind et al., 2009). All identifications were manually validated to reduce deconvolution errors and to eliminate false identifications. The NIST 14 GC–MS library was also used to cross-validate the spectral matching scores obtained using the Agilent library and to provide identifications of unmatched metabolites. These spectral scores were used for downstream metabolomics analysis using MetaboAnalyst 5.0 (Pang et al., 2021) with sample normalization by median and log transformation of the data.

## 3 Results

### 3.1 Field soil moisture content by irrigation treatment, depth, and date

The different irrigation treatments impacted the soil moisture content, with a decrease as expected from most to least irrigated (**Supplemental Figure 2**). The decreases between treatments were confirmed to be significant (p < 0.05) through one-way analysis of variance (ANOVA) and Kruskal-Wallis tests, both for the overall dataset, as well as within each individual timepoint except for April non-control soils (i.e. samples taken prior to differential irrigation being put into place) (**Supplemental Table 1**). While gravimetric water content tended to decrease from surface soil to deeper layers (**Supplemental Figure 2**), ‘Depth’ only became a significant factor (p < 0.05) for water content in October (**Supplemental Table 1**). These results indicated that the irrigation treatment became significant as soon as the differential regimes were started (July and October), but depth only became significant as the treatment duration and water limitation in relevant treatments increased further.

### 3.2 Diversity of the soil and rhizosphere microbiomes

#### 3.2.1 Alpha-diversity

**Figure 2:**
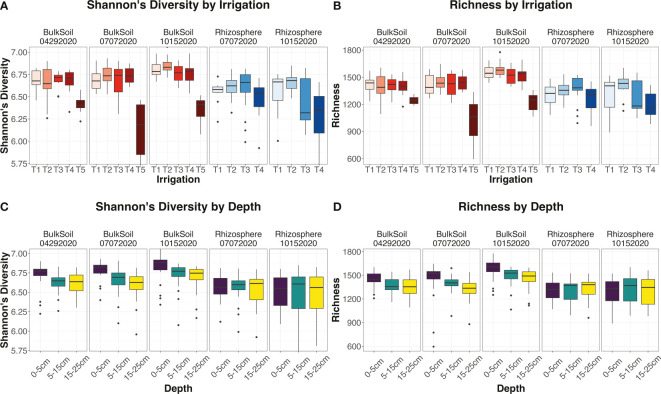
Trends for alpha-diversity metrics. **(A–D)** Boxplots for the alpha-diversity metrics Shannon’s diversity **(A, C)** or richness **(B, D)**, faceted by sample type (bulk soil or rhizosphere) and date (April, July, or October sampling dates). Trends are seen over irrigation regimes **(A, B)** or soil depths **(C, D)**. All boxplots were calculated using default parameters, where lower and upper hinges represent first and third quartiles, and all outliers fall outside these ranges.

After performing 16S amplicon sequencing on the soil and rhizosphere samples, there were 360 samples that passed the quality control checks. The resultant dataset encompassed a total of 18,546 operational taxonomic units (OTUs). One-way ANOVA tests (including Kruskal-Wallis tests) were used to determine how our experimental factors affected the alpha-diversity metrics ‘Shannon’s diversity’ and ‘richness’. In bulk soils, the different irrigation treatments within the treatment plots (T1 -> T4) had similar values for Shannon’s diversity and richness (**Supplemental Table 2**; **Figure 2**), while control unplanted soils (T5) were significantly lower, according to Tukey’s *post-hoc* tests (**Supplemental Table 3**). This indicated that differential irrigation regimes within treatment plots were not as influential on microbial diversity as simple presence/absence of irrigation. Interestingly, soils at intermediate moisture levels (T2 treatments) tended to have higher diversity values than T1 soils (**Figure 2**; **Supplemental Table 3**), which may implicate the full moisture treatment as being so high as to adversely affect microbiome diversity.

In contrast to soil, diversity levels in the rhizosphere showed significant differentiation by irrigation treatment at the final timepoint, according to Tukey’s *post-hoc* tests (**Supplemental Table 3**). Here, Shannon’s diversity and richness were both significantly (p < 0.05) lower in T4 rhizosphere samples than for the T1-T3 irrigation treatments (**Figure 2**; **Supplemental Table 3**). We had hypothesized that the rhizosphere would have a more attenuated response to irrigation than bulk soil, but the results here indicated the opposite: we observed that rhizosphere diversity was in fact affected more strongly by irrigation differences than bulk soil was.

Depth had a significant impact on alpha-diversity metrics in bulk soil (p < 0.05), but not in the rhizosphere (p = 0.909 and 0.943 for Shannon’s diversity and richness respectively) (**Supplemental Table 2**). Consistent with what has been seen in the literature (Fierer et al., 2003b; Eilers et al., 2012; Tripathi et al., 2019), Shannon’s diversity and richness both declined from surface to deeper soil layers in bulk soil (**Figure 2**; **Supplemental Table 2**), and the size of this effect increased over the course of the field season as irrigation differences increased. However, as depth did not significantly influence rhizosphere alpha-diversity metrics, this suggests that the plant maintains a consistent community diversity down the root length. In addition, the factor ‘Cultivar’ did not affect either diversity metric (**Supplemental Table 2**) in the rhizosphere – an unexpected finding, as plant genotype tends to have a strong effect on microbial communities, especially under drought (Orwin and Wardle, 2005). The cultivars may have been too closely related to show a measurable difference in diversity [plant genetic distance is positively correlated to rhizosphere dissimilarity (Naylor et al., 2017)].

As for the interplay between irrigation and depth, we found the interaction factor ‘Irrigation : Depth’, as well as the tripartite interaction ‘Date : Irrigation:Depth’, to both significantly affect alpha-diversity values in bulk soil (p < 0.05 for Shannon’s diversity and richness) (**Supplemental Table 2**). Investigating this interaction further, we found that in bulk soils, ‘Irrigation’ had a significant (p < 0.05) influence on Shannon’s diversity and richness in the surface 0-5 cm layer, and this effect was consistent across the entire field season. However, in deeper soil layers, ‘Irrigation’ was initially non-significant, but its effect sizes and significances increased over successive time points (**Supplemental Figure 3**).

Another aspect of the depth by irrigation effect was seen in how alpha-diversity metrics decreased from shallow to deeper soils. Specifically, the strength of this decrease was most pronounced in the moderately-irrigated T2 treatment, weaker in the lightly-irrigated T4 treated, and virtually nonexistent in the non-irrigated control T5 soils (**Supplemental Figures 3C, D**). These results indicated that the influence of depth on bulk soil microbial diversity was weakened as less soil moisture was applied. As for the rhizosphere, the decrease was not echoed in these samples, and the factor ‘Irrigation : Depth’ was not significant (**Supplemental Table 2**). This finding instead reiterated that rhizosphere communities responded to changes in irrigation in a uniform manner down the full length of the root (**Supplemental Figures 3A, B**).

#### 3.2.2 Beta-diversity

**Figure 3:**
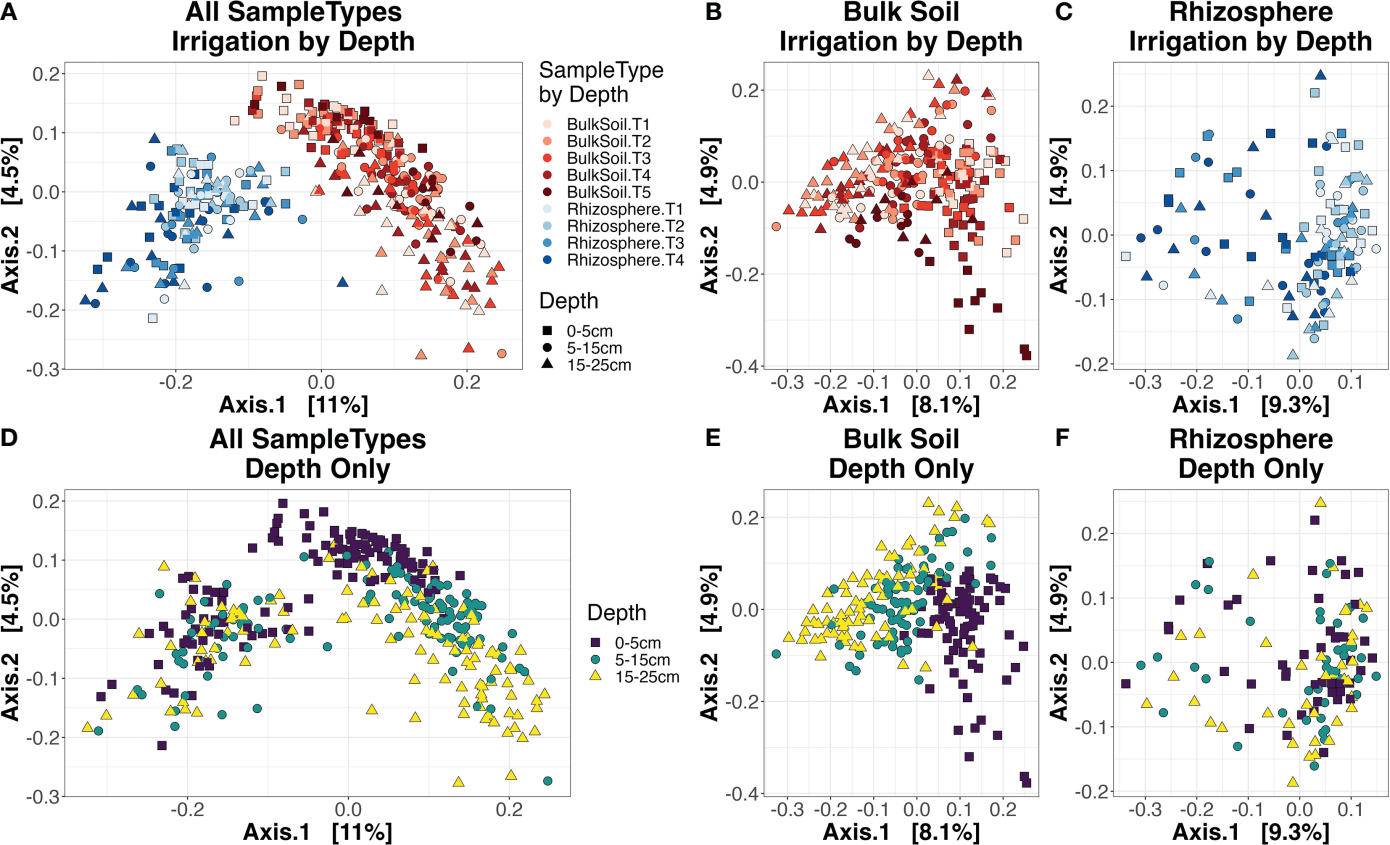
Ordination plots. Principal coordinate analysis (PCoA) for all samples **(A, D)**, bulk soil samples **(B, E)**, or rhizosphere samples **(C, F)**. Plots **(A-C)** are colored by the combination of sample type and irrigation **(A-C)** and the shape of each point corresponds to the depth (square for the top 0-5 cm layer, circle for 5-15 cm, and triangle for 15-25 cm). Plots **(D-F)** have their color and shape corresponding to depth only.

Dissimilarity between sample community profiles was estimated using the beta-diversity metric, Bray-Curtis distance. Bray-Curtis values, coupled with PERMANOVA (permutational analysis of variance) tests, were used to ascertain how strongly our experimental factors impacted community composition. On a Principal Coordinate Analysis (PCoA) ordination plot, samples primarily clustered by ‘SampleType’ (i.e., bulk soil or rhizosphere) along the primary axis (11% of variation), and by ‘Depth’ along the secondary axis (4.5%) (**Figures 3A, D**). According to PERMANOVA tests, the factor ‘Depth’ was highly significant (p < 0.001) for bulk soil samples, but not for rhizosphere (**Supplemental Table 4**; **Figures 3E, F**), similar to the results seen for alpha-diversity. While ‘Irrigation’ significantly (p < 0.001) influenced both sample types (**Supplemental Table 4**), samples did not tend to cluster strongly by irrigation treatment, whether for bulk soil (**Figure 3B**) or rhizosphere (**Figure 3C**). In the rhizosphere, the factor ‘Cultivar’ was just over the threshold of significance (p = 0.068) – however, the interaction factor ‘Irrigation : Cultivar’ was highly significant (p < 0.001) (**Supplemental Table 4**), implying that the rhizosphere microbiome’s response to irrigation shifts were partly tied to the host plant’s genotype.

The interaction of ‘Irrigation : Depth’ was again significant (p < 0.001) in bulk soils (**Supplemental Table 4**), indicating that irrigation impacted community composition differently depending on soil depth. However, beta-diversity metrics did not follow the same temporal patterns as seen for alpha-diversity indices. While at 0-5 cm and 5-15 cm the effect sizes of ‘Irrigation’ in soil became stronger and more significant over time, likely reflecting the heightened differences in water applied as the field season went on, the reverse trend was seen for the deepest 15-25 cm layer (**Supplemental Figure 3**). As for the rhizosphere, ‘Irrigation : Depth’ was again not significant (**Supplemental Table 4**), similar to the results seen for alpha-diversity.

### 3.3 Relative abundance patterns of microbiomes across irrigation treatments

**Figure 4:**
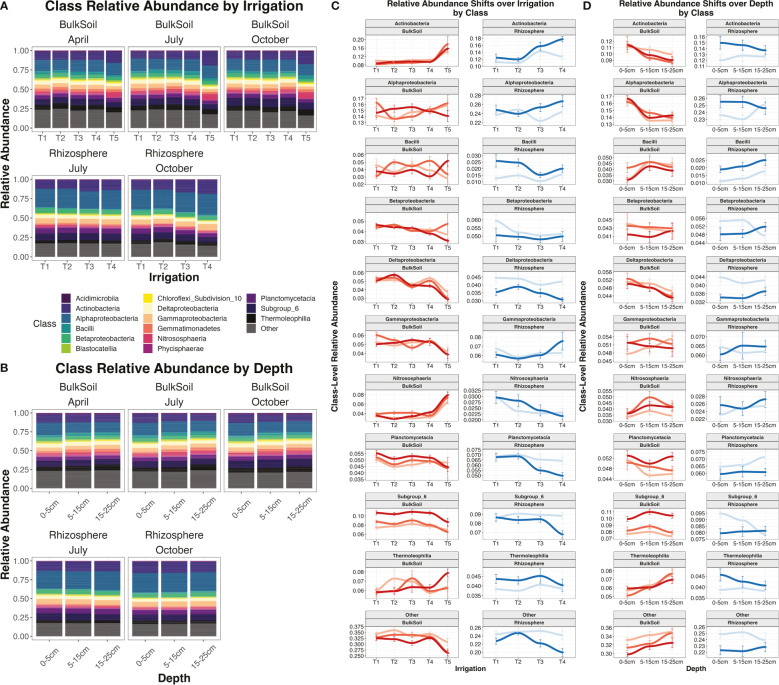
Relative abundance plots and relative abundances of individual classes. Relative abundance plot for irrigation treatments **(A)** or by depth **(B)** at the class level, segregated by date and sample type. Plots **(C)** and **(D)** show the individual relative abundances of each major class present in the dataset over irrigation **(C)** or over depth **(D)**. Colored lines indicate the combination of sample type (bulk soil or rhizosphere) and date (April, July, or October). Error bars are standard error.

Relative abundance plots were constructed to visualize microbial class-level abundance trends by irrigation regime (**Figures 4A, C**), or by depth (**Figures 4B, D**). Some microbial classes consistently increased or decreased across depth and/or irrigation levels, regardless of date or sample type. Meanwhile, other classes displayed more variable trends: i.e., the strength or directionality of their abundance trends changed based on sampling date or treatment. Polyserial correlation (Olsson et al., 1982) was used to calculate the statistical significance for the covariance of class abundances with sequential levels of irrigation (i.e. T1 -> T5) or depth (0-5 cm -> 15-25 cm) – i.e., are classes significantly increasing or decreasing in abundance as drought gets more severe, or as soils get deeper? Polyserial correlations were performed for all microbial classes, subsetting the overall dataset for each date by sample type combination (e.g. ‘July rhizosphere’), to determine how trends varied over time, as well as the distinctions between bulk soil and rhizosphere. All trends reported here are significant with p < 0.05.

For irrigation, the most pronounced trend was for the Actinobacteria class. Abundance of Actinobacteria was significantly positively correlated with the shift from T1 -> T4/T5 irrigation regimes (i.e increasingly dry soils) in all samples, apart from the July rhizosphere (**Supplemental Table 5**). Other classes that displayed consistent positive correlations with decreasing moisture included Cytophagia and Thermomicrobia (**Supplemental Table 5**). However, more often class abundances were negatively correlated with drought, most notably Betaproteobacteria, Deltaproteobacteria, and Planctomycetacia. Interestingly, some classes showed contrasting trends depending on sample type. The Nitrosphaeria class was correlated with irrigation in all samples, but while it increased in abundance as bulk soils got drier, in rhizosphere it decreased in abundance in drier samples (**Supplemental Table 5**). Likewise, Gammaproteobacteria showed the opposite trend, being negatively correlated with drought in bulk soil but positively in the rhizosphere (**Supplemental Table 5**).

The abundances of multiple microbial classes were significantly correlated with soil depth. However, in contrast to irrigation, depth trends by class were more distinct between bulk soil and rhizosphere. In bulk soil, 9 classes had a significant positive correlation with depth (i.e. increased in deeper soils) while another 9 had a negative correlation. However, in rhizosphere samples, only 2 classes were significantly positively correlated with greater depth, while another 2 were negatively correlated with depth (**Supplemental Table 5**), again implicating the rhizosphere as maintaining a more consistent community composition over depth than seen for bulk soil. The most consistent trends in bulk soil included significant decreases with depth for Alphaproteobacteria, Cytophagia, and Holophagae, and significant increases in Gemmatimonadetes and Thermeolophilia (**Supplemental Table 5**; **Figure 4D**). Interestingly, certain depth trends shifted over time. Actinobacteria and Deltaproteobacteria showed significant declines with depth in July and October, but not in April, while Planctomycetacia showed a significant decline with depth in April only (**Supplemental Table 5**).

**Figure 5:**
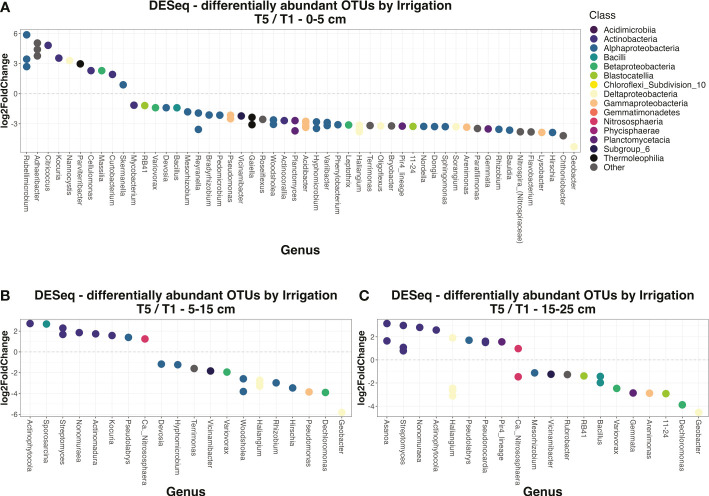
Differentially enriched OTUs by irrigation regime in soils. DESeq analyses were performed on the soil dataset to determine the OTUs that were significantly (adjusted p-value < 0.05) differentially expressed by irrigation regime (specifically the T5 *vs*. T1 division). Individual DESeq analyses were done for the top 0-5 cm layer **(A)**, the middle 5-15 cm layer **(B)**, and the bottom 15-25 cm layer **(C)**. The level of enrichment is given by log-2-fold change, where a positive value indicates enrichment in T5 treatments and negative in T1 treatments. OTUs are colored by their class, and their x-axis position corresponds to which genus they belong to. OTUs without a genus-level assignment were excluded from this figure.

To better investigate the irrigation by depth effect, soil and rhizosphere datasets were separated into subsets for each depth, then polyserial correlation analysis was performed to determine how many classes were responded to irrigation for each of the three soil layers. For bulk soils, the irrigation effect was strongest in the top layer, where there were 11 classes that were significantly correlated with changes in irrigation. Meanwhile, in the deeper two layers there were only 5 and 4 classes, respectively (**Supplemental Table 6**). Furthermore, the effect sizes and significances of class-irrigation correlations were generally lower in deeper layers than the top layer (**Supplemental Table 6**). We built upon these results by going down to the OTU level, employing DESeq analyses to determine how many OTUs were significantly (adjusted p-value < 0.05) differentially enriched between irrigation extremes (T5 *vs*. T1 treatments) at each of the three depths. Similar to the class-level results, far more individual OTUs were differentially enriched by irrigation at the 0-5 cm depth compared to the 5-15 cm or 15-25 cm depths in bulk soils (**Figure 5**). Taken together, these results reveal that irrigation poses more of a discriminating effect on community composition at the surface for bulk soil compared to deeper levels. As for the rhizosphere, while our previous results indicated that ‘Depth’ was not a strong discriminant on rhizosphere community composition overall (**Supplemental Table 4**, **Supplemental Figure 5**), we did observe some significant correlations between irrigation and abundances of individual classes, and these patterns changed with depth. In contrast to bulk soil, in rhizosphere there were actually far more significant correlations for class abundances with irrigation at the deeper 5-15 cm or 15-25 cm layers compared to the 0-5 cm layer (**Supplemental Table 6**).

Rhizosphere communities were significantly influenced by wheatgrass cultivar and these effects were strongest at the July timepoint (**Supplemental Figure 6A**). According to ANOVA tests, at July the tall wheatgrass line ‘Alkar’ had significantly elevated abundances for a number of classes (Acidimicrobiia, Betaproteobacteria, Blastocatellia, Chloroflexi subdivision 10, Gemmatimonadetes, Acidobacteria subgroup 6, Thermeophilia) compared to ‘Jose’. Jose was instead enriched for classes Alphaproteobacteria and Planctomycetacia relative to Alkar (**Supplemental Table 7**). However, at October no classes were significantly different between cultivars. Interestingly, the interactive effect of ‘Irrigation : Cultivar’ was stronger than that of ‘Cultivar’ alone (**Supplemental Table 4**), supporting our hypothesis that wheatgrass cultivars have distinct microbiome responses to water availability. For instance, under T1 treatments, Alkar was elevated for classes Acidimicrobiia and Deltaproteobacteria relative to Jose, but under T4, Alkar was instead elevated for a different set of classes including Gemmatimonadetes and Phycisphaerae (**Supplemental Figure 6B**, **Supplemental Table 7**). Similarly, DESeq analyses confirmed 92 differentially abundant taxa between irrigation extremes (T4/T1) in the Alkar rhizosphere, but only 1 for Jose (**Supplemental Figure 6D**). These results indicate that Alkar rhizospheres have a more pronounced response to irrigation changes than do Jose rhizospheres. Regardless of irrigation trends, however, the interactive effect ‘Depth : Cultivar’ was non-significant (**Supplemental Figure 6C**, **Supplemental Table 4**), which could be from cultivars having similar enrichment patterns down the length of the root, or simply due to ‘Depth’ having a weak influence in the rhizosphere.

### 3.4 Soil metabolomics patterns by depth and irrigation

We incorporated soil metabolomics datasets into our analyses to gain insights into how depth and irrigation impact the functional capacity of the soil microbiome. We specifically focused on soils from T1, T4, and T5 sites, which represented the irrigation extremes both within plots (T1 <-> T4) and for all soils (T1 <-> T5). We first determined which experimental factors were responsible for changes in metabolite profiles by conducting PERMANOVA tests on Bray-Curtis distance objects constructed for each of the April, July, and October datasets. Similar to the 16S data, irrigation had a more significant influence on soil metabolites at the July and October datasets (p = 0.004 and 0.005 respectively) compared to April (p = 0.039) (**Supplemental Table 8**). Likewise, depth significantly impacted the soil metabolite composition in July and October. However, while each factor was individually influential, the ‘Irrigation : Depth’ interaction was never significant (**Supplemental Table 8**), suggesting that the influence of irrigation on metabolite composition did not change across these soil depths. On PCoA plots, T1 and T4 samples grouped together, but both clustered apart from T5 (**Supplemental Figures 7A–C**), indicating that the difference between presence and absence of irrigation was the primary driver of sample differentiation. We did not see a strong depth effect on the metabolite profile, as samples within the same depth did not cluster more closely together than samples from other depths (**Supplemental Figures 7A–C**).

Next, we looked at the metabolite trends by irrigation and depth in greater detail. The criteria chosen to determine which metabolites were significantly different by T1 *vs*. T5 irrigation extremes were: (1) metabolites with a log-2-fold-change greater than 2 or less than -2 and (2) having an adjusted p-value (Benjamin-Hochberg correction) less than 0.05. There were 20 metabolites out of 210 total annotated metabolites that met these criteria for the April timepoint, 12 for July, and 23 for October (**Supplemental Table 9**). However, when examining for metabolites that were differentially abundant across depth extremes (0-5 cm *vs*. 15-25 cm), no metabolites met these criteria, regardless of timepoint. These results underscore that irrigation treatment, and not depth, was the primary influence on the soil metabolite composition. Annotated metabolites that were significantly enriched in the highly irrigated T1 treatment relative to non-irrigated T5 included amino acids (threonine, isoleucine, phenylalanine) and carbohydrates (tagatose, unknown carbohydrates). The only annotated metabolite that was enriched in the T5 bare soil treatment was the GABA precursor, 4-hydroxybutanoic acid (**Supplemental Table 9**).

### 3.5 Soil metatranscriptomics patterns by depth and irrigation

A subset of soil samples was taken to investigate the functional gene expression profiles between depth and irrigation extremes. Metatranscriptomes were obtained from 20 bulk soil samples, all from the October timepoint; these samples included replicates from the T1, T4, and control T5 irrigation treatments, and from both 0-5cm and 15-25cm depths. Transcripts were annotated against the EggNOG database to acquire KEGG functional information, including gene names and pathways from BRITE hierarchies.

**Figure 6:**
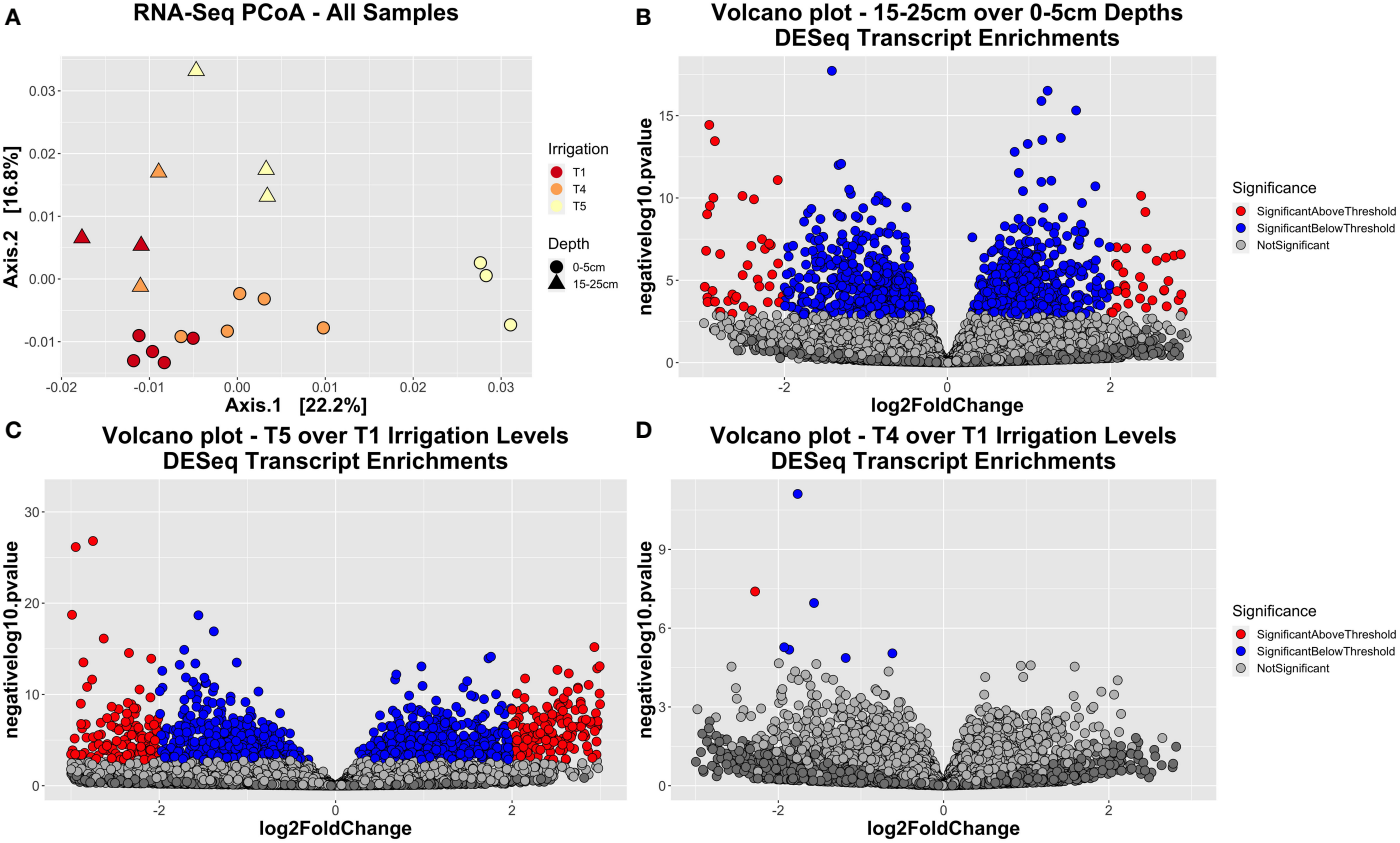
RNA-seq dataset sample separation and enrichment trends. **(A)** Principal coordinate ordination (PCoA) plot generated using Bray-Curtis dissimilarity, showing how samples segregate by their irrigation level (red = T1, orange = T4, yellow = T5) or depth (circle = surface soil, triangle = deep soil). **(B–D)** Volcano plots showing the enriched transcripts either between the two depth extremes **(B)**, the two irrigation extremes T5 *vs*. T1 **(C)**, or the two in-plot irrigation extremes T4 *vs*. T1 **(D)**. Enriched transcripts were calculated using a DESeq analysis. Transcripts with a p-value less than 0.01 are colored blue, except those that also have an absolute fold-change greater than 2, which are colored red.

Using this dataset, we first aimed to determine whether our experimental factors significantly influenced the metatranscriptome dataset. To do so, we constructed Bray-Curtis dissimilarity objects for DESeq-normalized transcript abundance. Through PERMANOVA, we found that the factors ‘Irrigation’, ‘Depth’ (both p < 0.001), as well as the interaction factor of ‘Irrigation : Depth’ (p = 0.021) were significant and respectively explained 15.4%, 22.1%, and 11.9% of variation (**Supplemental Table 10**). On a PCoA plot, samples clustered together by both irrigation and depth, although more strongly by depth (**Figure 6A**). We also observed functional diversity to be higher in bulk soils from lower depths (15-25 cm) compared to surface soils (0-5 cm), although the number of unique KO’s was lower (**Supplemental Figures 8A, C**).

Results from DESeq analysis indicated there was a wide array of KEGG Orthologs (KO’s) that had significantly different expression levels between surface and deep soils (**Supplemental Figure 9A**; **Figure 6B**), as well as between the irrigation extremes T1 *vs*. T5 (**Supplemental Figure 9B**; **Figure 6C**). To investigate the types of functions that were enriched, relative abundance plots were constructed to visually inspect pathway abundance trends by depth or irrigation (**Supplemental Figure 10**). Concurrently, a combination of ANOVA and Tukey’s *post-hoc* tests were used to ascertain if read counts for a given pathway were significantly influenced by experimental factors (Irrigation, Depth, Cultivar) - and if so, what the directionality was of this effect [**Supplemental Table 11**]. Of the 20 most abundant pathways, most of these pathways had significantly (p < 0.05) lower read counts in subsurface soils compared to surface soils, indicating bacteria had lower overall metabolic activity in deeper soils. Such pathways included ‘Biosynthesis of amino acids’, ‘Biosynthesis of secondary metabolites’, ‘Carbon fixation pathways in prokaryotes’, ‘Carbon metabolism’, ‘Citrate cycle’, ‘Glycolysis/gluconeogenesis’, ‘Glyoxylate and dicarboxylate metabolism’, ‘Metabolic pathways’, and ‘Oxidative phosphorylation’, among others (**Supplemental Figure 10A**, **Supplemental Table 11**). By contrast, not one of these 20 major pathways were significantly enriched in the deeper soils. With respect to irrigation level, a select few pathways were significantly (p < 0.05) elevated as soils got drier, including ‘Butanoate metabolism’, ‘Purine metabolism’, and ‘Quorum sensing’. Pathways that were significantly depleted in drier soils included ‘Glycolysis/gluconeogenesis’, ‘Ribosome’, ‘RNA degradation’, ‘RNA polymerase’, and ‘Two-component system’ (**Supplemental Figures 10C, D**, **Supplemental Table 11**). As for the interactive effect of depth by irrigation, pathways that were significantly (p < 0.05) depleted for both deeper and drier soils included ‘C5-Branched dibasic acid metabolism’, ‘Cellular senescence’, and ‘Plant-pathogen interaction’ (**Supplemental Table 11**).

To complement these results, Fisher’s exact test was applied to determine which metabolic pathways were represented to a significantly greater extent in a particular subset compared to the full dataset. By depth, the most striking trends were enrichment in the 0-5 cm soils for 17 KEGG categories. These included ‘Metabolic pathways’, ‘Microbial metabolism in diverse environments’, and ‘Nitrogen metabolism’, various pathways for metabolism of simple carbon compounds (aromatic acids, butanoate, fructose and mannose, etc.), as well as pathways for degradation of toxic chemicals (xylene, toluene, benzoate, nitrotoluene, chlorocyclohexane and chlorobenzene) (**Supplemental Table 12B**). The 15-25 cm soils were enriched for 12 categories, including pathways such as ‘Glycosphingolipid biosynthesis’, ‘Biofilm formation’, and ‘Flagellar assembly’. As for irrigation trends, the non-irrigated control T5 samples were surprisingly enriched for a variety of metabolism-related pathways (‘Carbon metabolism’, ‘Microbial metabolism in diverse environments’, ‘Peptidoglycan biosynthesis’, ‘Nitrogen metabolism’, etc.) (**Supplemental Table 12C**). Meanwhile, the irrigated T1 samples were enriched for a greater diversity of pathways, but fewer related to bacterial functioning - those that were enriched included ‘Fatty acid metabolism’, ‘Biofilm formation’, and ‘Flagellar assembly’ (**Supplemental Table 12D**).

Finally, we aimed to determine the extent to which the interaction of ‘Depth : Irrigation’ affected metabolic pathway abundances, as we had already ascertained that this interaction factor significantly influenced functional profiles overall (**Supplemental Table 10**). Here, we performed indicator analysis to ascertain which pathways were significantly associated with a particular combination of depth by irrigation (for example, “0-5cm_by_T1”). We found that there were 10 pathways that were indicators of “0-5cm_by_T1”, including functions such as “C5 Branched dibasic acid metabolism” and “Cellular senescence”, and 17 for “15-25cm_by_T1”, such as “ABC transporters”, “Pentose phosphate pathway”, “Sulfur metabolism”, and “Degradation of aromatic compounds” (**Supplemental Table 13**). For the intermediate T4 irrigation, there were only 6 indicators for the 15-25cm depth and none for the 0-5 cm depth, suggesting that there is little unique functionally for this level of irrigation and instead the greatest discrepancies in functioning are provoked by the irrigation extremes of T1 *vs*. T5. The T5 treatments showed a strikingly uneven distribution of enriched pathways between the two depths: the “0-5cm_by_T5” had 64 pathways that were indicators, many related to metabolism of carbon compounds, while the deeper “15-25cm_by_T5” level had none (**Supplemental Table 13**).

## 4 Discussion

In this work, we examined how soil moisture influences the soil microbiome and its functional potential at different soil depths. This knowledge is key to understanding how changes in soil moisture - which are predicted with climate change (Naylor et al., 2020b) - will influence soil ecology. Although the effects of soil depth on the resident microbiome have been previously investigated (Jiao et al., 2018; Brewer et al., 2019; Tripathi et al., 2019), as have the effects of irrigation (Bouskill et al., 2016; Preece and Peñuelas, 2016; Naylor et al., 2017), the combination of these two factors have rarely been studied in the context of soil microbiome composition (Potthoff et al., 2006) or functioning (Fierer et al., 2003a). An advantage to our study was that, because our field site was located in an arid environment, we were able more precisely to control the amount of water added to the soil by irrigation. In addition, because our field trial was planted with two varieties of tall wheatgrass, a potential bioenergy feedstock, we incorporated analyses of the influence of plant variety, combined with differences in soil moisture, on the soil microbiome at different depths. Finally, because seasonality is known to have a strong influence on the soil microbiome (Cruz-Martínez et al., 2009; Shigyo et al., 2019), we took into account this source of variability by sampling at the peak growing season in July as well as after the second harvest in October.

### 4.1 Diversity and abundance trends by depth and irrigation

Our results supported our main hypothesis pertaining to the depth by irrigation effect: that irrigation had more of an impact on the microbiome at the surface compared to deeper soil layers – although, these results were only consistently true for bulk soil and not rhizosphere (**Supplemental Figure 3**). Indeed, irrigation significantly affected the abundances of roughly twice as many bacterial classes, and four times as many OTUs, in the surface soil compared to either of the two deeper soil layers (**Supplemental Table 6**). The weaker effects of irrigation in deeper soils may have been because subsoil microbes are better insulated from environmental fluctuations in water supply (Dungait et al., 2012; Yan et al., 2017). In addition, taxa from lower soil depths inhabit a more nutrient-limiting microenvironment (Jobbágy and Jackson, 2001), and may be better adapted to deal with stress, meaning that their communities will likely be more resistant to changes in the environment (Brewer et al., 2019). However, over the course of the growing season we observed that the effects of irrigation in deeper soil layers generally became stronger and more significant. This may reflect how treatment severity was heightened over time, to the point that later in the season, irrigation treatment effects became strong enough to impact the microbes inhabiting deeper soil layers. Similarly, over the field season we observed changes in how depth impacted the microbiome: for example, Actinobacteria and Deltaproteobacteria decreased in abundance with depth across the season, but this effect was much stronger in later timepoints compared to the initial April timepoint. If treatment severity heightens over time, and deeper soils are impacted more and more, this will in turn have repercussions for which soil microbes are able to persist long-term at these levels.

Another explanation for our results could be that in deeper soils, microbes are slower to respond to changes in irrigation due to their life-strategies. As mentioned, deeper soils are more nutrient-limited (Jobbágy and Jackson, 2001). Nutrient-limited soils are more favorable for the slow-growing oligotrophs than faster-growing copiotrophs [which in turn are favored at the soil surface where labile carbon substrates are more abundant (Fierer et al., 2007)]. Consistent with previous studies (Seuradge et al., 2017; Sun et al., 2018a; Tripathi et al., 2019), we found that many oligogrophic taxa, including Gemmatimonadetes and Thermeolophilia were higher in relative abundance in deeper soils. Meanwhile, copiotrophic taxa, including Alphaproteobacteria, Cytophagia, and Holophagae, decreased with depth (**Supplemental Table 5**; **Figure 4D**). As for their life-strategies, Thermoleophilia are known to be associated with phosphorus limitation (Cui et al., 2018), and Gemmatimonadetes with low nitrogen (Cederlund et al., 2014). In particular, members of the Gemmatimonadetes have been shown to grow slowly and to be associated with nutrient and moisture limitation (DeBruyn et al., 2013) - again, typical traits of oligotrophs. By contrast, some members of the Alphaproteobacteria and Cytophagia have been associated with copiotroph life strategies (Fierer et al., 2007), explaining their depletion with depth. Not much is known about Holophagae, but its enrichment in deeper soils could be due to its members being largely anaerobic (Fukunaga and Ichikawa, 2014), which is favored in the more oxygen-poor deeper soils (Naylor et al., 2022). In the deeper soils, many of the enriched taxa included genera known to degrade complex polysaccharides through extracellular enzyme production; these included *Streptomyces* (Celaya-Herrera et al., 2021), *Paenibacillus* (Ashraf et al., 2017), and *Cohnella* (Fathallh Eida et al., 2012). Other enriched taxa in deeper soils (*Actinophytocola, Actinomadura*) (**Supplemental Table 14**) have previously been associated with resource-scarce environments such as deserts (Kurapova et al., 2012; Sun et al., 2018b). Taken together, we found that deeper soils were enriched for taxa associated with oligotrophic lifestyles, which could explain why deeper soil communities were more resistant to differences in soil moisture. We do acknowledge that fluctuations in moisture are likely to be less pronounced in deeper layers compared to surface layers, so it may transpire that the effect of irrigation was simply lessened in deeper soils rather than it being an intrinsic property of the subsoil microbiome.

Similar to depth, droughted soils have previously been shown to enrich for oligotrophs (Naylor and Coleman-Derr, 2018). However, irrigation trends here did not necessarily follow the same copiotroph-oligotroph distinction as we found with depth. We did see that the copiotrophic classes Beta- and Deltaproteobacteria decreased in drier soils, which is consistent with what would be expected. Meanwhile, Actinobacteria was strongly enriched under drought, which is consistent with previous findings (Bouskill et al., 2013; Chodak et al., 2015; Hartmann et al., 2017), where their survival under drought has been linked to their thicker cell membrane preventing against desiccation, as well as their ability to degrade recalcitrant carbon sources as labile substrates become scarcer (Naylor and Coleman-Derr, 2018). However, this group contains members that can utilize labile carbon, recalcitrant carbon, or both, and has been labeled as either copiotrophic or oligotrophic (Morrissey et al., 2016; Ho et al., 2017), so we cannot solely link Actinobacterial enrichment to their metabolic proclivities. Similarly, Cytophagia increased with drought while Chloroflexi decreased. Cytophaga is associated with a copiotrophic life-strategy (Fierer et al., 2007), while Chloroflexi is a known cellulose degrader (Pepe-Ranney et al., 2016), a trait of oligotrophs, so in this case our findings are inconsistent with our hypothesis. Indicator analysis likewise found contrasting results: there were oligotrophic genera enriched under drought [e.g. *Citricoccus* (Hayano-Kanashiro et al., 2011), *Actinophytocola* (Sun et al., 2014), *Cellulomonas* (Hatayama et al., 2013), and *Pedobacter* (Senechkin, 2013)], but also copiotrophic genera [*Massilia* (Ofek et al., 2012), *Kocuria* (Tashyrev and Prekrasna, 2014), and *Asanoa* (Xu et al., 2011; Niemhom et al., 2016)] (**Supplemental Table 15**). Together, these results suggest other reasons beyond carbon metabolism for a microbial class’s prevalence under different moisture regimes. One possibility is that greater stress levels lead to greater presence of relic DNA (i.e. from dead microbes) in the soil, leading to artificially inflated abundance estimates. However, as relic DNA degradation rates are predicted to be similar between species, we would expect that community profiles to not be substantially different between relic-containing and relic-depleted DNA (Lennon et al., 2018). In particular, the differences between those two types of DNA were minimal for communities under pulse drying-rewetting disturbance (Kittredge et al., 2022), which is fairly analogous to the experimental conditions of this study. Another possibility could be changes in physical and chemical factors in the soil, induced by drying, affect the microbiome. For instance, more water could mean that salt and oxygen concentrations go down, that water-soluble nutrients and carbon substrates become more available through diffusion, or that microbe-microbe interactions increase in frequency (Naylor and Coleman-Derr, 2018). Given this nuance, the changes in the soil induced by moisture fluctuations can therefore not be simplified to simple correlations between taxa abundances and soil water content. Furthermore, in our PERMANOVA analyses, the significant experimental factors generally explained a relatively small proportion of variation, leaving a high residual. This indicates that there are other unmeasured factors that influence the microbiome. Future analyses on soil depth and the microbiome could incorporate measurements of soil chemical and physical factors, to get a more complete picture of the various direct and indirect ways the microbiome is affected.

Interestingly, the class Nitrosphaeria were enriched under drought in soils but depleted in the rhizosphere, while the class Gammaproteobacteria had the opposite trend. This suggests that in certain cases the plant can circumvent the changes in community dynamics that occur in the underlying soil. Nitrosphaeria represents a major ammonia-oxidizing archaeal group that can form biofilms (Kerou et al., 2016). Biofilm formation could present an advantage in droughted bulk soils, but Nitrosphaeria may be outcompeted in the carbon-rich rhizosphere by fast-growing bacteria (Taffner et al., 2019). Gammaproteobacteria, by contrast, was negatively correlated with drought in soil but positively in roots (**Supplemental Table 5**). This may reflect the increased proportion of carbon allocated for rhizodeposition under drought (Preece and Peñuelas, 2016) favoring the fast-growing microbes within this class, such as members of genus *Pseudomonas*.

### 4.2 Abundance trends in rhizosphere by irrigation and depth

The plants used in our field trial, tall wheatgrass, are deep-rooting perennial grasses (Nie et al., 2008). We found that the composition of the tall wheatgrass rhizosphere microbiome was surprisingly consistent along the different sampling depths, suggesting that the root is a strong selector of specific microbes irrespective of depth. The ‘rhizosphere effect’ is a well-known phenomenon (Bakker et al., 2013) in which the plant enriches for a targeted subset of the surrounding soil microbiome to inhabit root-associated compartments. The fact that community composition in the rhizosphere was generally the same down the length of the root (**Supplemental Figure 5**) implicates the plant’s enrichment effect as being strong enough to overpower most local variation in the starting soil inoculum. It is possible that, while our depth intervals were sufficient to evince a change in the bulk soil communities, they were still too shallow to see a comparable change in that of the rhizosphere, and we may have needed to sample deeper to see a real effect – however, the vast majority of root tissue did not extend past our lower limit of 25 cm depth at the time of sampling, precluding this as a viable alternative.

The irrigation-specific trends in the rhizosphere were similar to those seen in bulk soil (drought-linked enrichment for Actinobacteria and Cytophagia, and drought-linked depletion in Deltaproteobacteria and Nitrososphaeria) (**Supplemental Table 4**). With respect to Actinobacteria, in addition to the aforementioned physical properties that lead to be enriched in dry soils, their enrichment in rhizosphere under drought could be linked to their plant growth promoting abilities, including conferring drought stress tolerance to plants. For example, isolates of the Actinobacterial genus *Streptomyces* were shown to stimulate sorghum root growth under drought stress (Xu et al., 2018), and others can stimulate plant root growth through the enzyme ACC deaminase (Naylor and Coleman-Derr, 2018). Possessing these functions could explain why Actinobacterial enrichment was observed in the rhizosphere as well as in bulk soil. Alternatively, Actinobacteria could be increasing in relative abundance by their membrane physiology allowing them to resist the toxic effects of the reactive oxygen species produced by the plant under drought stress (Xu et al., 2018).

Interestingly, the rhizosphere microbiome displayed a stronger response to irrigation (**Supplemental Tables 2**, **4**) than the response seen in bulk soil. This is possibly a consequence of plants actively responding to decreases in moisture availability by recruiting a beneficial microbiome tailored for those conditions, as both wheatgrass cultivars used in this study have been bred to tolerate low-moisture conditions to improve their suitability as a biofuel feedstock on marginal lands. Alternatively, it is possible that less tolerant wheatgrass cultivars’ rhizosphere microbiomes fail to respond as strongly to irrigation changes, meaning they are less equipped to recruit beneficial microbes and thereby withstand drought stress. A better understanding of the substances that plant uses to recruit a beneficial microbiome could be achieved through metabolomics approaches. For example, mass spectrometry could be performed on wheatgrass rhizosphere soils from both control and drought conditions, the major differentially abundant metabolites identified, then these metabolites could be applied to unplanted soil to see changes in microbial abundance trends. In this way, patterns of plant exudate release could be experimentally linked to microbial recruitment. However, it was beyond the scope of this paper to perform such an analysis, given our limited amount of rhizosphere soil was only sufficient to perform amplicon analysis.

Despite rhizosphere communities being statistically similar across different depths, we did observe non-significant trends towards slightly stronger irrigation effects for deeper rhizosphere communities: there were more differentially abundant microbial classes between irrigation extremes for rhizosphere communities at 5-15 cm and 15-25 cm when compared to the surface layer. These differences are likely not attributable to differences in moisture availability between the different soil layers, as relative water contents at T1 and T4 levels were largely comparable regardless of depth (data not shown). In our case, the finding of more differentially abundant rhizosphere taxa at greater depths may be attributable to the fact that root age decreases with depth, and nascent root tips are more actively involved in root exudation (Canarini et al., 2019) – thus, modulation of root-associated microbial communities (Preece and Peñuelas, 2016) should be more pronounced at deeper levels where root tips are located. Similarly, differences in rhizosphere community composition and/or diversity between root depths have been seen in *Salvia lyrata* (Dickey et al., 2020), in bunchgrass fields (Kuske et al., 2002), and in mixed populations of *Ferula* species (Wang et al., 2018). Such spatial gradients are proposed to be attributable to an uneven release of carbon and other nutrients down the length of the root (Hinsinger et al., 2009). While overall we did not observe a strong depth by irrigation effect in the rhizosphere, it would be of interest to investigate additional plant species (perhaps with deeper roots) or perhaps to heighten the differences between irrigation treatments, to see how effects of this interaction in the rhizosphere might be increased.

### 4.3 Abundance trends between wheatgrass genotypes

Another question we had the opportunity to address was whether there were genotype-dependent differences in the rhizosphere microbiome. As the Alkar cultivar is adapted to the local environment (i.e. eastern Washington), we posited that it would be better positioned, when faced with drought stress, to recruit a new microbiome in a sympatric environment. Here, we found the interaction between ‘Irrigation’ and ‘Cultivar’ to be highly significant in rhizosphere samples, which was reflected in the cultivar-specific irrigation effects on the rhizosphere microbiome. For instance, under the driest field irrigation treatment, several microbial classes and OTUs had significantly higher abundances in the Alkar rhizosphere than in the Jose rhizosphere. Several of the Alkar-enriched microbial classes, including Gemmatimonadetes and Phycisphaerae, have previously been associated with arid soils (DeBruyn et al., 2011), and with drought stress (Ullah et al., 2019). There are a couple of possible explanations for this finding. Jose (which was bred in New Mexico) may be more resilient to drought stress and less reliant on recruitment of native microbes for mitigation of drought stress than Alkar. Alternatively, Jose may be less effective at recruitment of indigenous members of the soil microbiome in a comparatively foreign environment (Lau and Lennon, 2012; Zolla et al., 2013). Yet another possibility is that Jose may have already recruited a beneficial microbiome earlier in development and thus does not need to further alter its microbiome upon imposition of drought stress. We also observed that the cultivar effects were strongest at July compared to October, with far more significantly differentially enriched taxa in July. This finding may be attributable to the temperature. The addition of heat to drought stress can result in compounding variables with more pronounced microbiome responses than drought alone (Wipf et al., 2021). This may explain why we observed more cultivar differences in July, compared to October when the temperature was cooler.

### 4.4 Soil transcriptomics and metabolomics trends by depth and irrigation

We hypothesized that both deeper and drier soils would enrich for more oligotrophic communities, and these changes would be reflected in patterns in transcriptomic and metabolomic datasets. Furthermore, we hypothesized that the interaction between depth and irrigation would be significant for both of these omics datasets. Here, we confirmed through PERMANOVA that ‘Depth’, ‘Irrigation’, and their interaction all significantly influenced variation across the transcriptomic dataset (**Supplemental Table 10**). Our findings are consistent with previous studies finding a significant influence of depth (Yu et al., 2017) or soil moisture content (Brockett et al., 2012) on functional composition – although to our knowledge, the combination of these two factors has not yet been studied with respect to expression profiles. The results here indicate that there is a substantial interaction between the two factors that should be considered in future transcriptomic studies. That being said, we did not observe any significant interaction between depth and irrigation with respect to soil metabolomic dataset, which may due to lower resolution for this dataset compared to transcriptomics. Alternatively, while both depth and irrigation were significant, the combination of these treatment effects may have been too weak to elicit a measurable interactive effect in the metabolite profile.

While we did not observe any significantly differentially abundant soil metabolites between depth extremes, in the transcriptomic dataset there were noticeable patterns by depth. Firstly, there were fewer unique KOs in subsoils compared to surface soils (**Supplemental Figure 8C**), and fewer enriched pathways (**Supplemental Figure 9B**), consistent with previous findings (Goberna et al., 2005; Chen et al., 2015). Soil metabolic diversity is expected to decline in deeper soils for a number of reasons – for instance, as carbon sources are more limited (Goberna et al., 2005). Here, the metabolic pathways that were elevated in surface-level soil metatranscriptomes included those for metabolism of simple carbon compounds, like aromatic acids, or fructose and mannose, as well as those for degradation of xenobiotics (xylene, toluene, nitrotoluene, etc.). In the latter case, such chemicals are commonly found in herbicides, and the capacity for their degradation is consistent with soil microbes’ role in xenobiotic degradation (Jia et al., 2007; Schwarz et al., 2018). Our field site (including the bare soils) was treated with herbicides (2,4-D) prior to planting, thus observations of enriched pathways for degradation of intermediates of herbicide chemicals was not surprising. As for metabolism of carbon compounds, relative depletion of these pathways in deeper soils is understandable, due to the scarcity of labile carbon substrates in these layers relative to the surface (Rumpel and Kögel-Knabner, 2011). These findings are generally consistent with our expectation that surface soils would be more resource-replete and support a copiotrophic life-strategy. That being said, we did not observe a concomitant increase in expression of pathways for utilization of complex polysaccharides (i.e., an oligotrophic life-strategy) in deeper soil layers as might be expected based on previous research (Zhang et al., 2017). Given that our field site was originally established in the spring of 2018, the deeper soil layers at the time of sampling in the summer and autumn of 2020 may not have had time to accumulate substantial SOM to enrich for the corresponding microbial breakdown processes. We did observe an enrichment for expressed pathways involved in cell structures or membrane synthesis with greater depth, including glycosphingolipid biosynthesis, biofilm formation, and flagellar assembly. These functions may be related to the harsher conditions at depth – for instance, biofilms are conducive to microbes communicating with one another and synergistic interactions (Yang et al., 2021), whereas flagella could be used for microbes to actively seek out substrates in nutrient-poor subsoils (Rumpel and Kögel-Knabner, 2011).

With respect to how irrigation differences affected functional profiles, a greater number of expressed pathways were enriched in wetter soils than drier soils, which is consistent with reports that moisture is correlated with microbial activity (Hueso et al., 2012). As with deeper soils, a pathway enriched in wetter soils was flagellar assembly, which makes sense as flagella aid microbes in moving through water. In the drier soils, there was, however, an elevation of transcripts involved in carbon and nitrogen metabolism. There are a number of activities that these trends in increased metabolism could be related to, such as increased synthesis of osmolytes to maintain cellular turgor in drier soils (Welsh, 2000), or alternatively that wetter soils were more anoxic and thus less conducive to microbial metabolism. With respect to the metabolomics dataset, we observed certain amino acids and carbohydrates to be significantly lower in drier soils, which could be reflective of the higher levels of metabolism depleting them from the surrounding environment. The one annotated metabolite enriched in drier soils was the GABA precursor, 4-hydroxybutanoic acid. GABA itself is implicated in microbial spore germination for species such as *Bacillus megaterium* (Dhakal et al., 2012), so its enrichment in drier soils may make sense if bacteria are forming spores under drought stress. Apart from that, pathways for glycolysis/gluconeogenesis and fatty acid metabolism were both depleted in drier soils, as was biofilm formation, which is surprising given that biofilms are an essential means by which microbes maintain hydration in desiccated environments (Yang et al., 2021). It is possible that water levels were simply too low to allow for biofilm synthesis, as the extracellular polysaccharides that make up biofilms are ~97% water (Flemming et al., 2016).

Previous research has indicated there are overlapping effects for how soil depth and drought affect microbial functioning – for example, in both deeper and drier soils, overall microbial activity goes down, degradation of complex organic matter becomes more important, and microbes that persist are enriched for stress-related functions such as dormancy or sporulation. There is not a universal correlation, however – for instance, anaerobic processes are generally more prevalent in deeper soils than they are in drier ones (Naylor and Coleman-Derr, 2018; Naylor et al., 2022). Relatively few studies have examined the influence of different moisture availability on the soil microbiome at different depths, and those that have generally found little to no interactive effect of depth by irrigation on community composition or diversity (Engelhardt et al., 2018; Hao et al., 2020). Furthermore, to our knowledge this study represents the first time this interactive effect has been investigated with respect to microbial functional profiles. Our results here yielded interesting patterns with respect to this interaction: specifically, we found a somewhat more equitable distribution of enriched pathways between the two depths at the T1 irrigation regime as compared to non-irrigated T5. The shallow soils under the T5 regime included enriched pathways for carbon cycling, including metabolism of butanoate, propanoate, nitrogen, methane, among other compounds. The deeper soils under the T1 treatment were enriched for a number of similar pathways. Compared to the 0-5 cm T1 soils, these two soil sample sets were either deeper (15-25 cm T1) or drier (0-5 cm T5), and likely represent somewhat harsher soil conditions. Therefore, one possibility for the observed enrichment trends is that microbes need to elevate expression of these metabolic pathways to maintain carbon flow. We observed almost no enriched pathways for the deepest, driest soil (15-25 cm T5), which could be because the conditions would be harsh to the point where most active microbial expression is repressed. Another possibility for these trends could be that the T1 soils at 0-5 cm instead had lower expression for most pathways – if these soils were comparatively waterlogged, this would promote anaerobic conditions and repress many pathways for carbon metabolism. In this scenario, the other two soils are closer to what basal levels of microbial expression would look like – especially as these soils receive fairly little irrigation historically, so endogenous microbes would be adapted to a desiccated environment. Future soil experiments could incorporate complementary analyses (for example, quantitative PCR or measuring enzymatic activity levels) so as to investigate these functional trends in greater resolution.

## 5 Conclusion

Our study contributes to the emerging field by demonstrating that there is a significant interactive effect of depth by irrigation in soils, in which the influence of differential irrigation is strongest at surface levels, requires more time and/or a more severe treatment to substantially impact deeper soils, and is only very weakly present in the rhizosphere. We showed that the influence of depth outweighs that of irrigation in bulk soil, whereas in the rhizosphere, the influence of irrigation is stronger than that of depth. Transcriptomics results pointed to a narrowing of metabolic complexity with increasing soil depth. Additionally, we also observed a significant interactive effect between depth and irrigation for these functional profiles, where differences between wet and dry soils were far more pronounced at the surface layer than the deepest layer. Further research targeted at elucidating the types of microbes found at different layers and the metabolic pathways they actively express, particularly with regards to biogeochemical pathways such as carbon and nitrogen cycling, will be of great significance upon not only soil microbiome studies, but also broader avenues such as computational modeling for global biogeochemical cycling and greenhouse gas fluxes (Jenkinson and Coleman, 2008).

## Data availability statement

The data presented in this study are deposited in online repositories. Sequence data are deposited in DataHub (see https://data.pnl.gov/group/7/nodes/dataset/34944 for metatranscriptomics, and https://data.pnl.gov/group/7/nodes/dataset/34943 for 16S amplicon data). Metabolomics data is available on MassIVE, accession number MSV000090655.

## Author contributions

DN performed the experiments, conducted bioinformatic analyses, and wrote the manuscript. KN and MS assisted with field sample collection. SC, CN, JT performed metabolomics processing and annotation. SF maintained the field site. RD and RM processed the transcriptomics dataset. RM, KH, and JJ reviewed and edited the manuscript. KH and JJ obtained funding for the study. All authors contributed to the article and approved the submitted version.

## Supplementary material

Supplementary Figure 1 Field layout. The field was set up as three replicate blocks: in total, there were 60 plots in a randomized block design with three replications and two subsample variety plots per irrigation. To obtain one combination of irrigation regime x cultivar per replicate block, the sites 31 - 60 were sampled from (excluding ‘Empty’ columns and substituting plot 18 for 33 to obtain a ‘T4’ + ‘Alkar’ sample in replicate block 1). Control soil samples were taken on the south side of the field site immediately outside of the plot (close to sites 45, 30, and 15 for control soil samples 1, 2, and 3 respectively).

Supplementary Figure 2 Kernel destiny estimation for gravimetric moisture content of soil. Relative water content was calculated by dividing the fresh weight of soil by the dry weight. Plot **(A)** describes the differences in water content by irrigation treatment at each timepoint, while plot **(B)** describes the differences by soil depth at each of the three timepoints.

Supplementary Figure 3 ANOVA variance trends for alpha-diversity. The percentage of variance explained by the factor ‘Irrigation’ for Shannon’s diversity (top row) or richness (middle row), according to ANOVA tests. Bottom row is significance of ‘Irrigation’ for Bray-Curtis distance according to PERMANOVA tests. The y-axis shows the percentage of variance explained, and the shade darkness corresponds to the significance (‘*’ represents a p-value of 0.05 *-* 0.01, ‘**’ is p = 0.01 - 0.001, ‘***’ is p < 0.001).

Supplementary Figure 4 Trends for alpha-diversity metrics by irrigation, segregated by depth and sample type. Shannon’s diversity **(A)** and richness **(B)** trends by irrigation regime for the three depth layers in bulk soil (top row) and rhizosphere (bottom row). Shannon’s diversity **(C)** and richness **(D)** trends by irrigation for the three depths, in bulk soil (top row) and rhizosphere (bottom row).

Supplementary Figure 5 Relative abundance plots and relative abundances of individual classes. Relative abundance plot at the class level faceted by ‘sample type by date’ and depth, with irrigation treatments as the x-axis. Only the top 15 most abundant classes are shown here.

Supplementary Figure 6 Relative abundance and enrichment trends for rhizosphere samples. Relative abundance trends between cultivars for rhizosphere sample, segregated by date only **(A)**, by date and irrigation **(B)**, or by date and depth **(C)**. Below **(D)** is a DESeq plot showing the differentially abundant OTUs between irrigation extremes (T4 *vs*. T1 treatments) in either Alkar rhizosphere (left) or Jose rhizosphere (right). Both dates are included in this analysis. Points are colored depending on which class they belong to (see legend in top right).

Supplementary Figure 7 PCA and Volcano plots for metabolomics samples, April-October. MetaboAnalyst was used to analyze trends in the metabolomic dataset. Ordination plots were constructed for the metabolomic datasets from each of three dates (which were processed separately and thus could not be directly compared). Samples were colored by irrigation, and shapes were used to denote depth (A-C). Volcano plots were made from the same datasets (D-F), to visualize the enriched metabolites at each timepoint. Metabolites enriched in wet soils are colored red, those enriched in dry soils are colored blue.

Supplementary Figure 8 Diversity of the RNA-seq dataset. Boxplots of Shannon’s diversity of the transcriptomics dataset are shown segregated by depth **(A)** and irrigation **(B)**. Richness plots for the same factors are seen in **(C)** and **(D)**.

Supplementary Figure 9 iPATH visualization of enriched pathways based on depth **(A)** and irrigation **(B)**. DESeq was used to generate lists of KEGG orthologs that were significantly differentially enriched between the two depth **(A)** or irrigation extremes **(B)**, further subsetting based on which KO’s had a shrunken log-fold change of >1.0 and an adjusted *P* value of <0.05. Then, visualization of KO’s on a microbial metabolism map was accomplished using iPath 3.0. For **(A)**, red pathways reflect the KO’s enriched in deeper soils, blue pathways for those in enriched in shallower soils. For **(B)**, red pathways indicate those enriched in dry T5 soil, blue for those enriched in wet T1 soil.

Supplementary Figure 10 Relative abundance trends for BRITE Pathways. Relative abundances for counts of transcripts, where the transcripts were colored based on which of the 20 most abundant BRITE pathways they belonged to. Abundances are segregated by individual sample and grouped by either depth **(A)** or irrigation **(C)**, or else samples are consolidated to show the differences between the depth extremes **(B)** or the three irrigation level extremes **(D)**.
