# Supplementary figures and images for: Interactive effects of depth and differential irrigation on soil microbiome composition and functioning
Source: Front Microbiomes. 2023 Mar 2;2:1078024. doi: 10.3389/frmbi.2023.1078024 (PMC12993636; doi:10.3389/frmbi.2023.1078024)

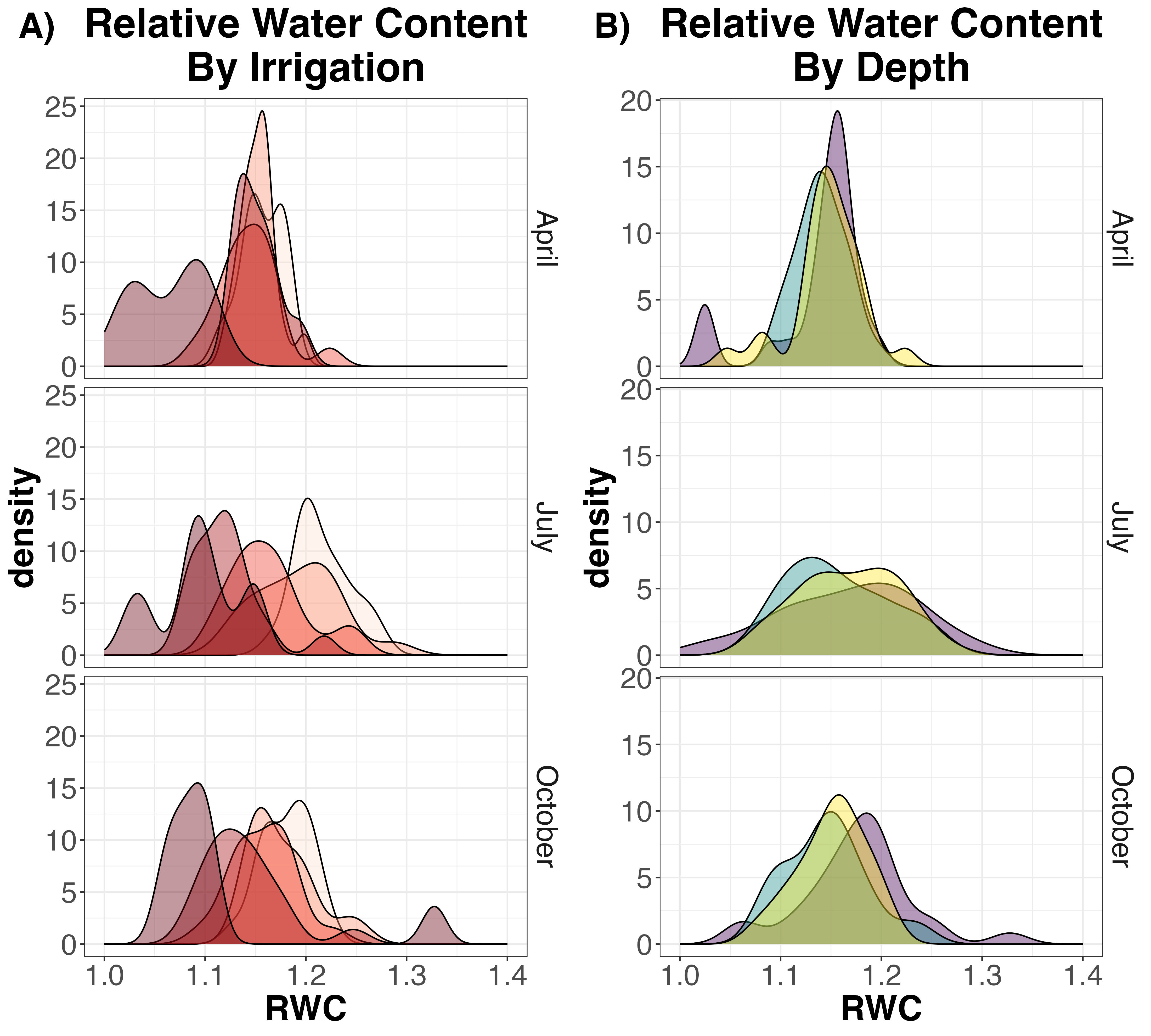

Supplement: Supplementary Figure 2 — Kernel destiny estimation for gravimetric moisture content of soil. Relative water content was calculated by dividing the fresh weight of soil by the dry weight. Plot (A) describes the differences in water content by irrigation treatment at each timepoint, while plot (B) describes the differences by soil depth at each of the three timepoints. [file Image_2.jpeg]

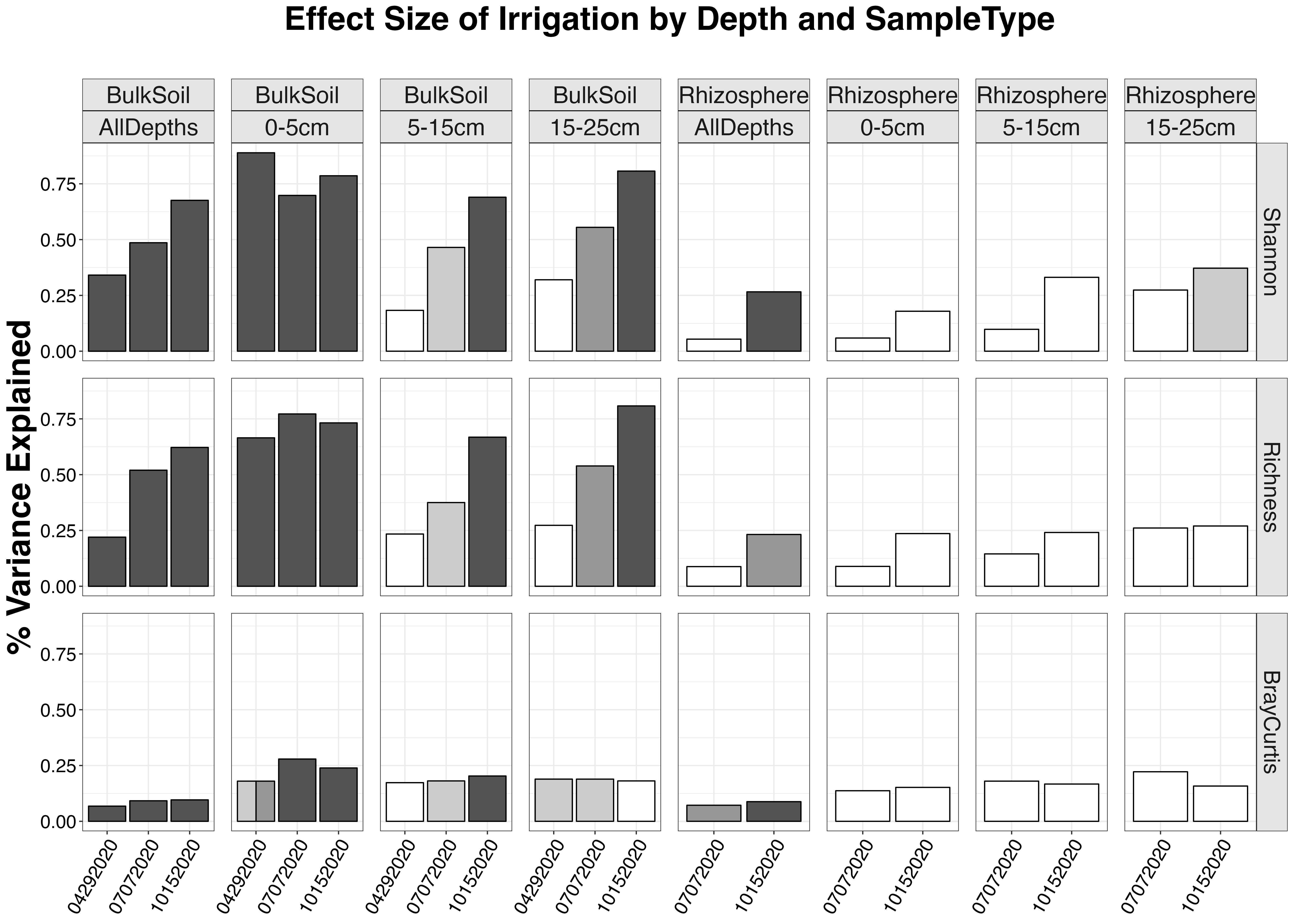

Supplement: Supplementary Figure 4 — Trends for alpha-diversity metrics by irrigation, segregated by depth and sample type. Shannon’s diversity (A) and richness (B) trends by irrigation regime for the three depth layers in bulk soil (top row) and rhizosphere (bottom row). Shannon’s diversity (C) and richness (D) trends by irrigation for the three depths, in bulk soil (top row) and rhizosphere (bottom row). [file Image_4.jpeg]

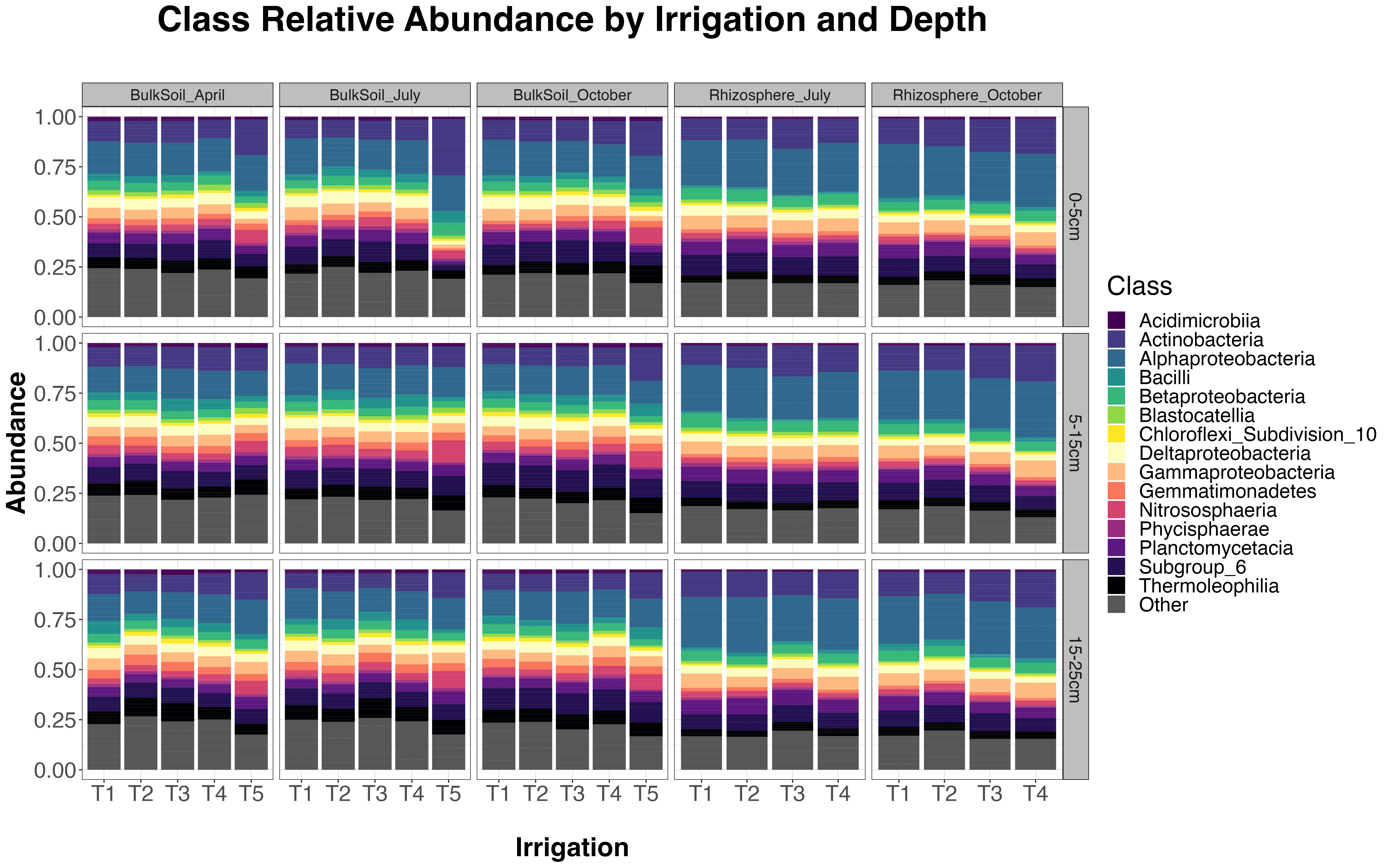

Supplement: Supplementary Figure 5 — Relative abundance plots and relative abundances of individual classes. Relative abundance plot at the class level faceted by ‘sample type by date’ and depth, with irrigation treatments as the x-axis. Only the top 15 most abundant classes are shown here. [file Image_5.jpeg]

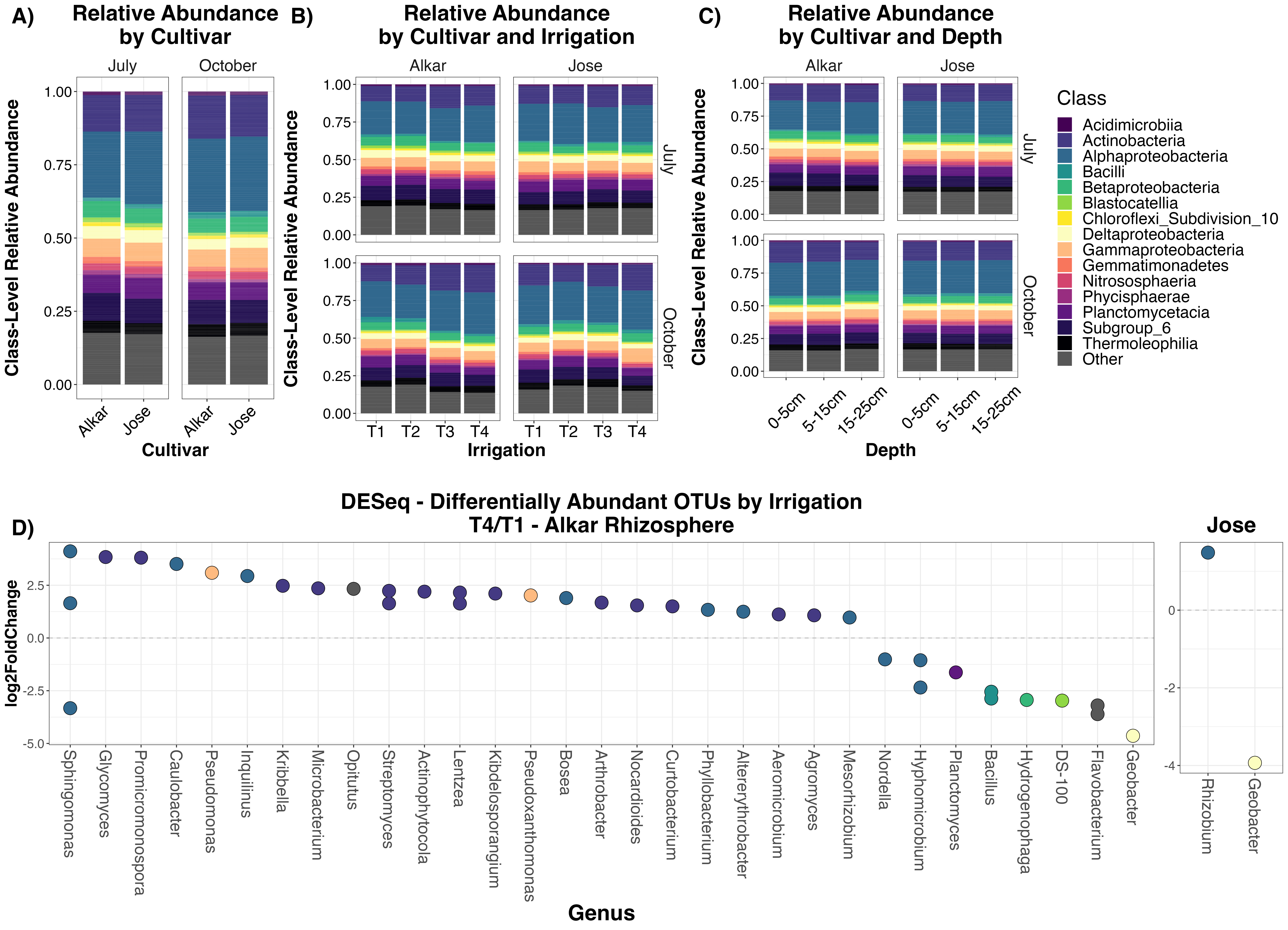

Supplement: Supplementary Figure 6 — Relative abundance and enrichment trends for rhizosphere samples. Relative abundance trends between cultivars for rhizosphere sample, segregated by date only (A), by date and irrigation (B), or by date and depth (C). Below (D) is a DESeq plot showing the differentially abundant OTUs between irrigation extremes (T4 vs. T1 treatments) in either Alkar rhizosphere (left) or Jose rhizosphere (right). Both dates are included in this analysis. Points are colored depending on which class they belong to (see legend in top right). [file Image_6.jpeg]

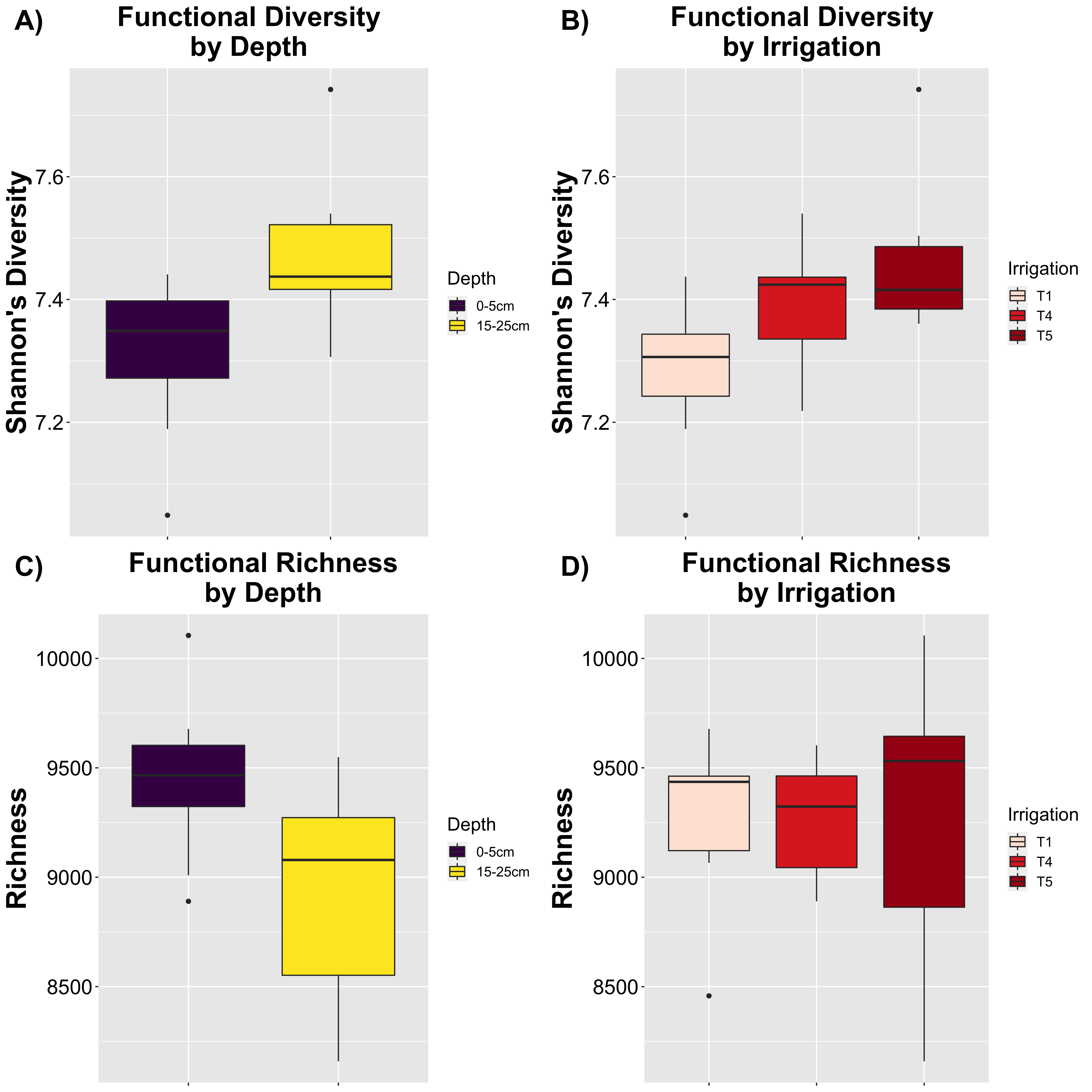

Supplement: Supplementary Figure 8 — Diversity of the RNA-seq dataset. Boxplots of Shannon’s diversity of the transcriptomics dataset are shown segregated by depth (A) and irrigation (B). Richness plots for the same factors are seen in (C) and (D). [file Image_8.jpeg]
